# Insights into the Transcriptional Reprogramming in Tomato Response to PSTVd Variants Using Network Approaches

**DOI:** 10.3390/ijms23115983

**Published:** 2022-05-26

**Authors:** Katia Aviña-Padilla, Octavio Zambada-Moreno, Gabriel Emilio Herrera-Oropeza, Marco A. Jimenez-Limas, Peter Abrahamian, Rosemarie W. Hammond, Maribel Hernández-Rosales

**Affiliations:** 1 Centro de Investigación y de Estudios Avanzados del I.P.N Unidad Irapuato, Irapuato 36821, Mexico; octavio.zambadam@cinvestav.mx; 2 Department of Crop Sciences, University of Illinois at Urbana-Champaign, Urbana, IL 61801, USA; 3 Center for Developmental Neurobiology, Institute of Psychiatry, Psychology, and Neuroscience, King’s College London, London WC2R 2LS, UK; gabriel.herrera_oropeza@kcl.ac.uk; 4 Centro de Investigación en Computación, Instituto Politécnico Nacional, Mexico City 07738, Mexico; marcojimenez@ciencias.unam.mx; 5 USDA, Agricultural Research Service, Beltsville Agricultural Research Center, Beltsville, MD 20705, USA; peter.abrahamian@usda.gov

**Keywords:** regulation of gene expression, master transcription factors, microproteins, plant-pathogen bioinformatics, biological networks analysis, systems biology, non-coding RNAs, interactome

## Abstract

Viroids are the smallest pathogens of angiosperms, consisting of non-coding RNAs that cause severe diseases in agronomic crops. Symptoms associated with viroid infection are linked to developmental alterations due to genetic regulation. To understand the global mechanisms of host viroid response, we implemented network approaches to identify master transcription regulators and their differentially expressed targets in tomato infected with mild and severe variants of PSTVd. Our approach integrates root and leaf transcriptomic data, gene regulatory network analysis, and identification of affected biological processes. Our results reveal that specific bHLH, MYB, and ERF transcription factors regulate genes involved in molecular mechanisms underlying critical signaling pathways. Functional enrichment of regulons shows that bHLH-MTRs are linked to metabolism and plant defense, while MYB-MTRs are involved in signaling and hormone-related processes. Strikingly, a member of the bHLH-TF family has a specific potential role as a microprotein involved in the post-translational regulation of hormone signaling events. We found that ERF-MTRs are characteristic of severe symptoms, while ZNF-TF, tf3a-TF, BZIP-TFs, and NAC-TF act as unique MTRs. Altogether, our results lay a foundation for further research on the PSTVd and host genome interaction, providing evidence for identifying potential key genes that influence symptom development in tomato plants.

## 1. Introduction

Viroids are plant specific parasites that cause significant losses in agronomically important crops. They are composed of non-coding, single-stranded, circular RNA (246–491 nt) of low molecular weight without a protein or lipid membrane [1]. The mechanism of systemic infection consists of hijacking the machinery of the host cell by interacting with cellular factors to achieve replication [2]. The proposed pathogenesis mechanisms may be regulated by the direct interaction between viroid genomic RNA, or its replication intermediates, and the host genome [3]. Environmental factors, such as photoperiod, temperature, and nutrient availability are crucial for viroid infection and may determine whether host plants are asymptomatic or exhibit a wide range of mild to severe symptoms. Symptom development is an outcome of a complex plant-pathogen interaction as a result of alterations to cell processes related to viroid RNA-mediated genetic regulation.

Genetic regulation is a highly coordinated process that comprises concerted events by multiple layers of interplay among DNA, RNA, and proteins. Plant response to pathogen infection conditions involves a precise reprogramming of host transcriptional activity. Improvements in technology and in data availability have led to an interest in systems biology approaches in plants to characterize the molecular mechanisms and key genes relevant to a specific tissue or disease condition. Recent advances employing next-generation sequencing have led to better insights into the molecular biology of viroid-host interactions. Transcriptional profiling analyses have revealed that viroid infections have a global effect on plant gene expression. These studies include potato spindle tuber viroid (PSTVd) infection in tomato [4,5,6,7,8] and pepper [8], citrus exocortis viroid (CEVd) [9] and citrus viroid III (CVd-III) [10] infection in Etrog citron, peach latent mosaic viroid (PLMVd) infection in peach [11], hop stunt viroid (HSVd) in hop [12] and cucumber [13], and hop latent viroid (HLVd) and citrus bark cracking viroid (CBCVd) in hop [14]. These previous studies showed alterations in host genes linked to defense, stress response, hormone signaling, DNA-binding, and RNA metabolism, among other functions [4,5,6,7,8,9,10,11,12,13,14,15].

Diseases driven by viroids cause serious economic losses in plants, in particular in tomato (*Solanum lycopersicum* L.) [3,4,5,6,7,8]. Tomato is one of the most widely grown greenhouse vegetables in the world and has been used as a model for basic research in plant development [16,17]. In addition, it is one of the most profitable and widely consumed by its various products. In 2020, 251,687,023 tons were harvested [18,19]. A high-quality genome sequence for domesticated tomato has been assembled by the International Tomato Genome Sequencing Project and more than 34,000 proteins have been predicted, with functional descriptions assigned to 29,532 genes (http://solgenomics.net/organism/Solanum_lycopersicum/genome, accessed on 1 January 2022), which includes 1845 genes that code for transcription factors (TFs) [20].

TFs are central regulators of gene expression, and their role relies upon their capacity to specifically interact with DNA sequences and other proteins as part of transcriptional complexes modulating key features of organismal biology, including cell differentiation, tissue and organ development, responses to hormones and environmental factors, metabolic networks, and disease resistance, among others. TFs regulate the transcription of downstream targets with the interaction of co-regulators known as regulons [21,22].

Various studies have recently reported several deregulated TFs in tomato and pepper hosts in response to PSTVd infection, stressing that the specific role of each of them must be considered to clarify their link to disease phenotypes, [7,8]. Taking advantage of data expansion, we used publicly available transcriptome profiles from microarray technology of time-course studies of mild (M.) and severe (S23) PSTVd infections in leaf and root samples derived from tomato plants [7,8]. Results from these studies highlighted differences in gene expression profiles depending on the infection stage and the PSTVd strain.

The transition from vegetative growth to reproductive development requires gene network coordination, where TFs act as essential regulators of organ morphogenesis. Our study focused on those that could play a key role in the expression of genes involved in biological processes linked to viroid-induced symptoms. For that aim, gene regulatory networks are proposed to infer an interaction mechanism. We used 53 expression profiles generated from plants in three different PSTVd infection stages with both mild (M.) and severe (S23) variants. In this work, we identified Master Transcriptional Regulators (MTRs), particularly in the (basic helix-loop-helix) bHLH, MYB, and (ethylene-response factors) ERF superfamilies, that have been shown to have a relevant role in viroid infection [7,8]. In plants, these TFs regulate growth, development, and stress response [23,24,25,26,27,28,29,30].

Moreover, there is recent evidence for the formation of highly regulated protein complexes through protein-protein interaction domains, the disruption of which can have serious consequences for cell function. In this context, the formation of dimers and multimers can be disturbed by proteins known as microproteins (miPs) [31,32]. We have recently reported the role of miPs of the conserved bHLH superfamily, which has evolved in both the animal and plant kingdoms [33]. Microproteins regulate multidomain proteins at the post-translational level. They are analogous to microRNAs, having the capability to heterodimerize with their targets, causing dominant and negative effects. In plants, bHLH-TFs have recently been characterized as miPs involved in cell elongation, brassinosteroid events, and plant development [32,34,35].

Our study integrates network models of genes affected during viroid infection in tomato, leading us to propose mechanisms of host gene regulation involving MTRs and targets under their regulation, as well as potential specific miPs. Overall, our results delineate the highly specific and pivotal role that bHLH, MYB, and ERF MTRs play in coordinating gene regulatory networks in PSTVd-infected tomato hosts. Strikingly, our analysis also highlights specificity at a post-translational regulation level involving bHLH miP candidates linked to auxin-responsive factors (ARFs), jasmonate (JA), and brassinosteroid pathways (BR), with a distinctive signature for the tomato response to infection with the severe strain of PSTVd.

The analysis of plant-pathogen interactions is an emerging research field that is very important for productive agricultural systems. The selective pressures driving these processes are extreme. For instance, the increased virulence of a pathogen places selective solid pressure on the plant host to increase or modify specific aspects of its defense response. If this is successful, selective pressures increase on the pathogen for compensatory changes in its ability to overcome these defenses.

Pospiviroid is a genus of viroids that most commonly infects species of *Solanaceae*. It belongs to the family Pospiviroidae. PSTVd is the first viroid discovered and model of study of pospiviroid species. The replication of pospiviroids takes place in the nucleus and regulates the expression of key genes by a still unknown mechanism that involves the transcriptional reprogramming by host-encoded biomolecules. Identifying the key regulators, the interacting miPs, as well as the downstream differentially expressed genes will contribute to unraveling the pathogenesis mechanism responsible for the characteristic phenotypes shown in the susceptible infected plants.

## 2. Results

### 2.1. Gene Regulatory Networks Identify Specific bHLH, ERF, and MYB TFs That Act as Master Transcriptional Regulators in the Tomato Response to PSTVd Infection

**Figure 1:**
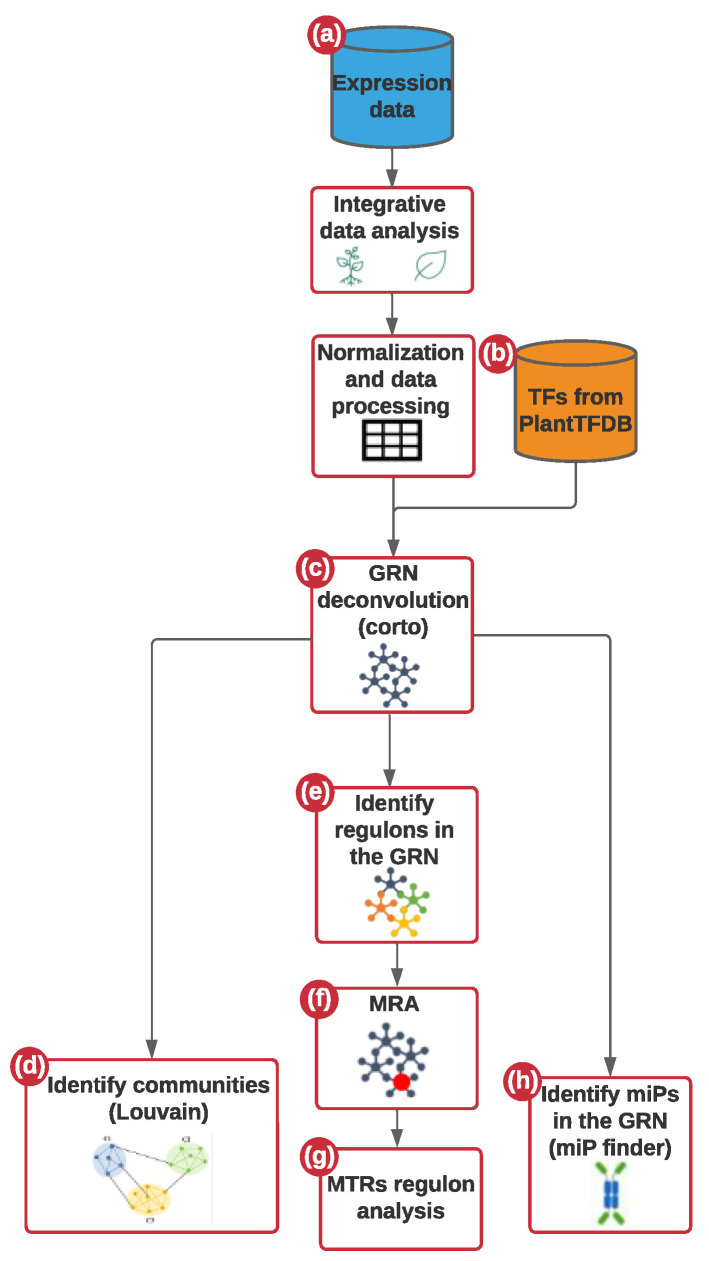
Pipeline for the omics approach. (**a**) First, microarray publicly available expression data of both control and infected leaves and roots were processed to obtain a unique expression matrix (**b**) The expression matrix and a list of tomato genome transcription factors from PlantTFD were used as input to (**c**) infer a network of transcriptional regulation employing Corto algorithm. (**d**) Then, biological communities were found in the network clustering genes by their metrics using the Louvain algorithm. From the obtained GRN (**e**) a regulon network was obtained by associating the expression level of the targets of all the transcription factors (**f**) Subsequently, the MRA algorithm was used to calculate the MTRs in the network (**g**) Once the MTRs are inferred we proceed to explore the functional role of them and their regulons. (**h**) Additionally, the bHLH-coding genes for miPs were assigned in the tomato genome. Among them, we identify those that are interacting in the GRN, and those that could be potential MTRs.

We used integrated transcriptome microarray data obtained from the NCBI Gene Expression Omnibus (GEO; GEO Overview-GEO-NCBI (nih.gov), accessed on 7 May 2021) for time-course analyses under three experimental conditions: Healthy Control (C.), PSTVd-Mild (M.), and PSTVd-Severe (S23) infections in roots (GSE111736) and leaf (GSE106912) [7,8] tissue. This dataset is the largest available for tomato viroid infection. We built a global gene regulatory network (GRN) integrating the three conditions above in which interactions represent the regulation of a TF to their target genes. The pipeline implemented in this study is described in Figure 1.

It has been observed that a number of large-scale transcriptional cascades behind complex cellular processes can be explained by the action of a relatively small number of MTRs [22,23,24]. The identification of the most crucial factors governing a determined phenotypic state is known as Master Regulator Analysis (MRA) [25]. We performed MRA to generate a robust readout not only of the changes of the transcript of each protein-encoding gene in the interactome but also to study its proximal functional network, [36]. In this study, MTR is defined as a TF whose regulon expression is significantly affected when comparing two conditions (i.e., A vs. B). MTRs can be inferred as therefore responsible for the transition from phenotype A to phenotype B [21].

The global GRN of the integrated samples is depicted in Figure S1. The MRA results showed that out of the 1845 annotated TFs in the tomato genome, 87 are MTRs modulating biological processes among the three different conditions (Figure S1).

In the C. vs. M. comparison, we identified seven significant MTRs, while, for the C. vs. S23 condition, we found 59 significant MTRs. On the other hand, when comparing M. vs. S23 the results indicated 21 significant MTRs (see Table S1). The largest difference in the plant phenotypes underlying the response to the aggressiveness of the viroid variant, was found in the C. vs. S23 comparison, with more than 60% of the significant MTRs.

The most significant MTRs identified in the C. vs. S23 condition were pivotal TFs, such as TCP-TFs (*Solyc01g008230.2*) and (*Solyc06g070900.2*), ZINC FINGER TF15 (*Solyc01g110490.2*), MADS-box (*Solyc01g087990.2*), BZIP (*Solyc01g111580.2*), and GRAS (*Solyc05g053090.1*) members. Despite the most significant factors being from different families, the TFs of the bHLH, MYB, and ERF families stand out in the MRA analysis. Notably, in the C. vs. S23 comparison, five members of the bHLH family SlbHLH011 (*Solyc01g111130.2*), bHLH130-like (*Solyc12g100140.1*), SlbHLH022 (*Solyc03g097820.1*), GBOF-1 (*Solyc06g072520.1*), and bHLH92 isoform (*Solyc09g083360.2*) are included. The following three MYB TFs were identified: MYB1R1 (*Solyc04g005100.2*), MYB16 (*Solyc02g088190.2*), and kua1 isoform x1 (*Solyc08g078340.2*). Seven ERFs were also identified: ERF-1a (*Solyc05g051200.1*), ERF_C_5 (*Solyc02g077370.1*), AP2/EREBP TF1 (*Solyc02g093130.1*), RAP2-12 (*Solyc12g049560.1*), PTI6 (*Solyc06g082590.1*), TSRF1 (*Solyc09g089930.1*), and ERF_A_2 (*Solyc03g093610.1*).

In the M. vs. S23 comparison, two bHLH-MTRs were identified: bHLH010, a cryptochrome-interacting basic-helix-loop-helix (*Solyc01g109700.2*), and MYC2, a JA-related TF (Solyc08g076930.1). Additionally, two MYB-TFs, kua1 isoform x1 (*Solyc08g078340.2*) and PHR1-LIKE 1 (*Solyc05g055940.2*), and two ERF TFs, AP2/EREBP TF1 (*Solyc02g093130.1*) and ERF_C_5 (*Solyc02g077370.1*), were identified.

In the C. vs. M. comparison, only an MYB-TF (*Solyc03g098320.2*) was predicted to function as an MTR. In total, seven bHLH, seven ERF, and five MYB condition-specific members were identified as MTRs in our study. We selected and focused on the members of those families to gain insight into their roles based on their representation and high specificity in the different treatment conditions analyzed.

### 2.2. Gene Communities in the bHLH, ERF, and MYB Interactomes Highlight Their Functional Significance in Specific and Shared Biological Processes

The identification of communities or modules can aid in the analysis of large biological networks by determining the biological systems that are coordinately regulated. In order to determine mechanisms of regulation in our global GRN, we used the Louvain community detection method that allows us to identify gene communities that could be participating together in particular biological processes [37,38]. For that purpose, we split our global GRN into subnetworks of the bHLH, MYB, and ERF MTR’s interactomes with their regulons.

#### 2.2.1. Communities in the bHLH Interactome

Our analysis revealed that in the interactome of the bHLH MTRs four biological communities were identified. See Appendix A for the communities’ descriptions, and Appendix B for all the functional enrichment analyses performed in this study. The first contained 2040 genes enriched in GO:0015979 photosynthesis (*p*-value = 0.0000015). A second community contained 1439 genes functionally enriched in KEGG:00660-C5-Branched dibasic acid metabolism (*p*-value = 0.002571), and KEGG:00290-Valine, leucine, and isoleucine biosynthesis pathways (*p*-value = 0.0276). The third community contained 877 genes linked to the KEGG:03010-Ribosome pathway, GO:0042254 ribosome biogenesis, and anthocyanin biosynthesis, (*p*-value > 0.000003–0.01352). Finally, in the fourth community which includes 870 genes, an enrichment in DNA-binding TF activity and specific mRNA binding was determined (*p*-value = 3.969 × 10^−2^).

#### 2.2.2. Communities in the ERF Interactome

Regarding the ERF subnetwork, ten gene communities with biological significance were identified. This is the interactome with the highest number of communities found participating in multiple biological processes. The community with the highest number of genes has 578 enriched in the GO:0045272-plasma membrane respiratory chain complex (*p*-value = 0.00254). Meanwhile, the second community comprises 280 genes that are enriched in protein self-association and an aromatic amino acid family biosynthetic process. A regulation of cellular biosynthetic process was identified (*p*-value 4.507 × 10^−2^ > 3.343 × 10^−2^). The third includes 268 genes enriched in the KEGG:04626-plant-pathogen interaction pathway (*p*-value = 0.00121). A community of 247 genes is enriched in the KEGG:00750-Vitamin B6 metabolism pathway (*p*-value = 0.03634), and 201 genes via protein heterodimerization activity, serine O-acyltransferase activity, and nucleosome organization and packaging (*p*-value = 0.03634). Two communities of 158 genes were found and are related to the KEGG:00660-C5-Branched dibasic acid metabolism (*p*-value = 0.0399) and ribosomal large subunit biogenesis. Those pathways are shared with the communities found in the bHLH TF interactome. In addition, 95 genes participate in a community involved in the regulation of the cell cycle (*p*-value = 9.041 × 10^−3^), 81 genes enriched in regulation of cell wall pectin metabolic process, self-proteolysis, positive regulation of response to stimulus, and positive regulation of MAP kinase activity (*p*-value = 4.659 × 10^−2^ > 2.800 × 10^−2^), while the last community comprises 71 genes that are enriched in the KEGG:00942-Anthocyanin biosynthesis pathway (*p*-value = 0.01998).

#### 2.2.3. Communities in the MYB Interactome

Finally, when performing the operation for the MYB TFs interactome, we identified five gene communities participating in additional processes. For instance, we found the following: 1852 genes in GO:0015979 photosynthesis (*p*-value = 2.8596 × 10^−10^); 1804 genes in cellular divalent inorganic cation homeostasis rRNA binding GO:0019843 (*p*-value = 0.01183); 1715 genes in the following biological processes: GO:0006950 response to stress, GO:0006952 defense response, and the KEGG:04626 Plant-pathogen interaction pathway (*p*-value 0.0024 > 0.0367); and 875 genes related to the GO:0031365N-terminal protein amino acid modification (*p*-value = 0.0327). The presence of a small community of 12 genes participating in amino acid and carboxylic acid transmembrane transport (*p*-value = 6.839 × 10^−3^) was also found.

In summary, our results revealed that bHLH, ERF, and MYB TFs regulate gene expression in communities associated with unique biological processes, such as the valine, leucine, and isoleucine biosynthesis, vitamin B6 metabolism, anthocyanin biosynthesis, amino acid, and other molecules transmembrane transport, KEGG pathways, as well as participating together in the modulation of conserved crucial molecular processes, such as the C5-Branched dibasic acid metabolism, ribosome, photosynthesis, plant-pathogen interaction, and defense response in the PSTVd variants infection.

### 2.3. bHLH MTRs Are Specific Modulators of Genes Involved in Metabolism, Ribosome, Light, and Plant Defense Processes

**Figure 2:**
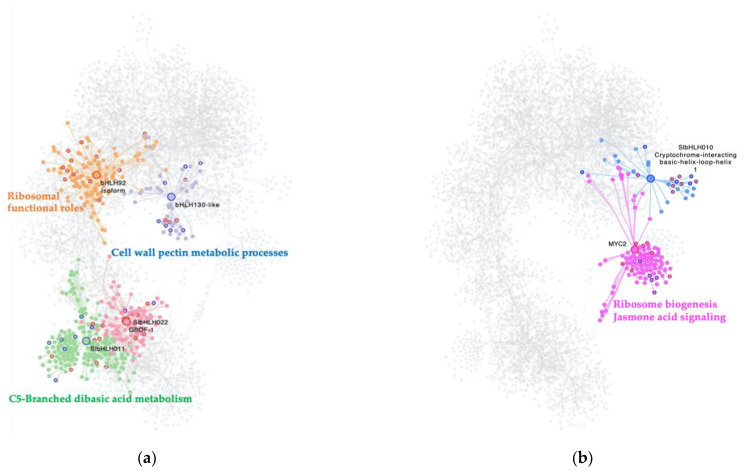
Interactome of the bHLH MTRs in the experimental conditions comparisons: (**a**) C. vs. S23 condition, (**b**): M. vs. S23 condition. Each node represents a gene, and the edges between nodes represent regulatory interactions between genes. The big, highlighted nodes with a specific color represent bHLH-MTRs and the colored perimeter of each MTR represents their NES value. Nodes of the same color surrounding these represent target genes regulated by an MTR, and the most differentially expressed genes in each regulon have colored perimeters: blue for downregulated, red for upregulated. Enriched biological processes of each regulon are presented in the same color as the regulon. In the comparison C. vs. S23 bHLH-MTRs are bHLH130-like (blue), bHLH92 (orange), SlbHLH011 (green), SlbHLH022 (cherry red), GBOF-1 (cherry red). Meanwhile, in the comparison M. vs. S23, bHLH-MTRs are MYC2 (pink) and SlbHLH010 (blue).

When analyzing the interactome of 32 bHLH TFs from the GRN, and their regulons, a total of 4411 nodes, and 7424 interactions, with the presence of seven MTRs were obtained. For the C. vs. S23 comparison, five unique members of this family were classified as MTRs, Figure 2a. The MRA results showed induction of their regulons: SlbHLH022 (*Solyc03g097820.1*); GBOF-1 (*Solyc06g072520.1*) with unknown functions; and bHLH92 isoform (*Solyc09g083360.2*) enriched in ribosome and ribonucleoprotein complex biogenesis functional roles, Table S1; Figure 2a. In contrast, the inhibition of regulons under the modulation of SlbHLH011 (*Solyc01g111130.2*), linked to C5-Branched dibasic acid metabolism, and bHLH130-like (*Solyc12g100140.1*), related to cell wall pectin metabolic processes, was identified in Table S1; Figure 2a.

In the comparison of the M. vs. S23, the relevance of two bHLH TFs was revealed in the MRA analysis. The induction of the MYC2 regulon (*Solyc08g076930.1*) is related to ribosome biogenesis and JA-signaling, while the repression of bHLH010 a cryptochrome-interacting basic-helix-loop-helix (*Solyc01g109700.2*) has no functional enrichment; however, this gene is related to triggering flowering in response to blue light [29] Figure 2b.

Strikingly, none of the bHLH-identified MTRs are shared between the C., M., and S23 treatment conditions. Overall, these results suggest that bHLH reprogramming of regulatory networks could be specific to PSTVd variant infection in its tomato host.

### 2.4. MYB MTRs Are Associated with Gene Regulation in Cell Division, Signaling, and mRNA Transcription

An interactome containing 39 MYB TFs from the GRN was generated to give deep insight and a better understanding of the role of this TF family in viroid infection and symptom development. The MYB interactome is composed of 4380 nodes with 7312 interactions among them.

**Figure 3:**
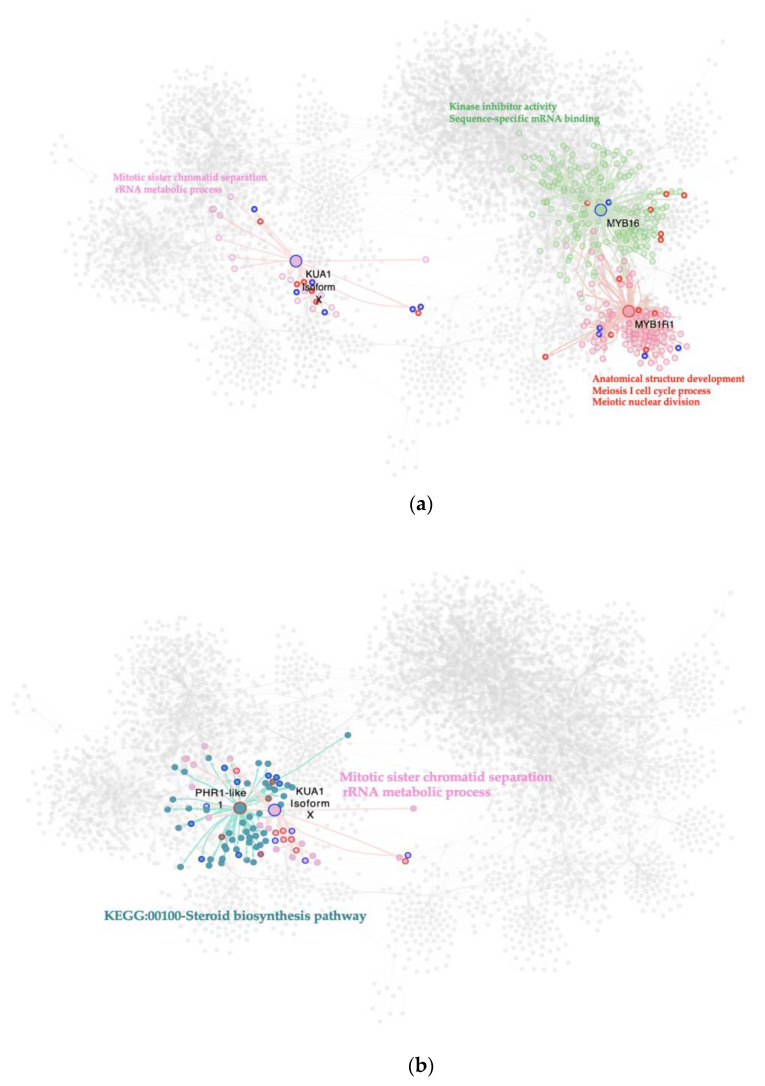

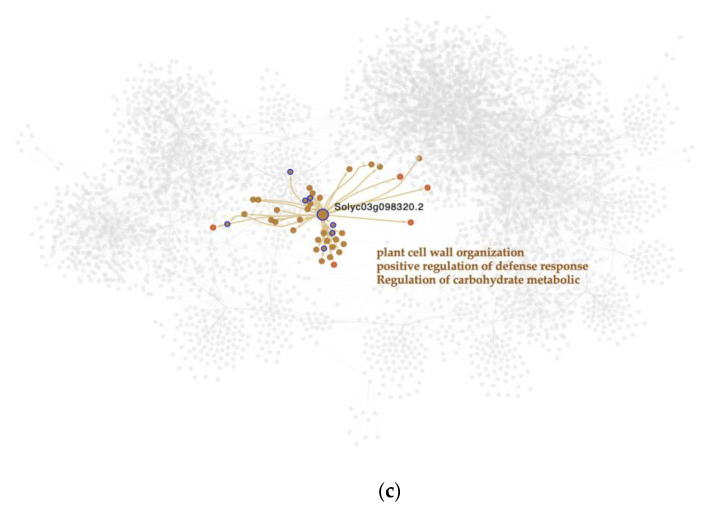
Interactome of the MYB-MTRs in the experimental conditions comparisons: (**a**) C. vs. S23 condition, (**b**) M. vs. S23 condition, (**c**) C. vs. M. condition. Each node represents a gene; the edges between nodes represent regulatory interactions between genes. The big, highlighted nodes with a specific color represent MYB-MTRs, while nodes of the same color surrounding these represent target genes regulated by an MTR. The most differentially expressed genes in each regulon have colored perimeters: blue for downregulation, red for upregulation. Enriched biological processes of each regulon are presented in the same color as the regulon. In the comparison C. vs. S23 MYB-MTRs are KUA1 (pink), MYB16 (green), and MYB1R1 (cherry red). Meanwhile, in the comparison M. vs. S23 MYB-MTRs are PHR1-like (aqua) and KUA1 (pink). For the M. vs. C. comparison, *Solyc03g098320.2* (brown) is a MYB-MTR.

Our MRA results show that for the C. vs. S23 comparison, a total of three MYB-TFs are MTRs (Figure 3a). Two modulate a lower expression of their regulons: MYB16 (*Solyc02g088190.2*) related to kinase inhibitor activity and sequence-specific mRNA binding, and kua1 isoform x1 (*Solyc08g078340.2*), which is enriched in the regulation of mitotic sister chromatid separation and rRNA metabolism. Meanwhile, upregulation of downstream gene expression of the MYB1R1 (*Solyc04g005100.2*) regulon is implicated in anatomical structure development and the meiosis I cell cycle process. The meiotic nuclear division was identified.

For the M. vs. S23 condition, two MYB TFs act as MTRs. Due to viroid response, a gene that encodes a kua1 isoform x1 (*Solyc08g078340.2*) represses the expression of its target genes, which are involved in the regulation of the mitotic cell cycle, while the PHR1-LIKE 1 (*Solyc05g055940.2*) regulon shows an increased expression, likely affecting the KEGG:00100-Steroid biosynthesis pathway where it participates (Figure 3b).

Notably, a member of this family was the only significant MTR found in the C. vs. M. strain comparison, Figure 3c. This MTR gene is *Solyc03g098320.2* which represses the expression of its downstream targets, which are enriched in plant cell wall organization, positive regulation of defense response, and regulation of carbohydrate metabolic biological processes.

### 2.5. ERF-MTRs Induce the Expression of Their Regulons as a Characteristic of the Tomato Response to the Severe PSTVd Infection

As for the other TF families of interest, an ERF-TFs subnetwork was built. This network contains 2475 nodes and 4075 interactions, being the smallest one of the three TF subnetworks.

**Figure 4:**
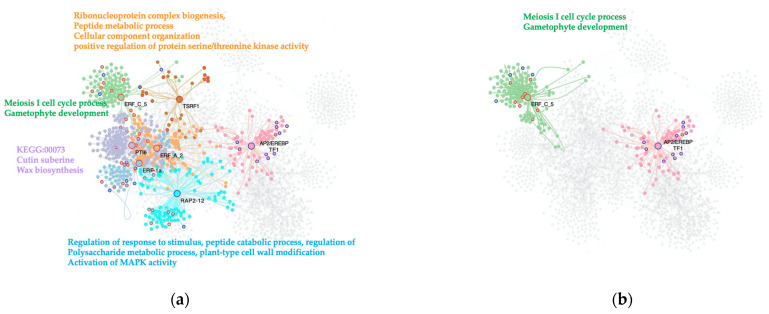
Interactome of the ERFs MTRs in the experimental condition’s comparisons. (**a**) C. vs. S23 condition, (**b**): M. vs. S23 condition. Each node represents a gene, the edges between nodes represent regulatory interactions between genes. The big, highlighted nodes with a specific color represent ERF-MTRs, while nodes of the same color surrounding these represent target genes regulated by an MTR. The most differentially expressed genes in each regulon have colored perimeters, blue for downregulation, red for upregulation. Enriched biological processes of each regulon are presented in the same color as the regulon. In the comparison C. vs. S23 ERF-MTRs are ERF_A_2 (orange), AP2/EREBP TF1 (pink), ERF_C_5 (green), PTI6 (purple), RAP2-12 (light blue), ERF-1a (blue) and TSR1 (brown). Meanwhile, in the comparison, M. vs. S23 ERF-MTRs are AP2/EREBP TF1 (pink) and ERF_C_5 (green).

The C. vs. S23 condition analysis determined seven genes of this family act as MTRs. Notably, the ERF-MTRs are abundant in the tomato response to the severe PSTVd variant. These results are in agreement with the previous PSTVd-transcriptomic study that indicates the expression of genes encoding ERFs, Pti4, Pti6, and dehydration-responsive element-binding proteins was altered, with most being upregulated [8]. The majority of the ERF-MTRs (five out of seven) induce the expression of their regulons: ERF-1a (*Solyc05g051200.1*); ERF_C_5 (*Solyc02g077370.1*)*;* PTI6 (*Solyc06g082590.1*); TSRF1 (*Solyc09g089930.1*); and ERF_A_2 (*Solyc03g093610.1*). Two repress the expression of their target genes: AP2/EREBP TF1 (*Solyc02g093130.1*) and RAP2-12 (*Solyc12g049560.1*), Figure 4a.

One annotated biological process induced in this comparison was resistance to fungi mediated by ERF-1a (*Solyc05g051200.1*), which also is a key integrator of ethylene and JA-signals in the regulation of ethylene/JA-dependent defenses, as with TSRF1 (*Solyc09g089930.1*). In keeping with this, the meiosis I cell cycle process and gametophyte development are enriched terms in the regulon induced by the ERF_C_5-TF (*Solyc02g077370.1*), as well as the KEGG:00073- Cutin, suberine, and wax biosynthesis synaptonemal complex organization linked to the PTI6 (*Solyc06g082590.1*). Other enriched biological processes found in the upregulated regulons are the ribonucleoprotein complex biogenesis, peptide metabolic process, cellular component organization or biogenesis, and GO:0071902 positive regulation of protein serine/threonine kinase activity by the ERF_A_2-MTR (*Solyc03g093610.1*). The downregulated pathways linked to ERF-MTRs in our comparison were regulation of gene expression by stress factors and by components of stress signal transduction pathways by AP2/EREBP TF1 *(Solyc02g093130.1*), and regulation of response to stimulus, peptide catabolic process, regulation of polysaccharide metabolic process, and plant-type cell wall modification, as well as activation of MAPK activity RAP2-12 (*Solyc12g049560.1*).

When analyzing the M. vs. S23 comparison, two genes were selected as MTRs for the transition among phenotypes: AP2/EREBP TF1 (*Solyc02g093130.1*) and ERF_C_5 (*Solyc02g077370.1*). AP2/EREBP has been predicted as a transcriptional activator that binds to the GCC-box pathogenesis-related promoter element. This MTR may be involved in the regulation of gene expression by stress factors and by components of stress signal transduction pathways. In our analysis, AP2/EREBP TF1 reduced the expression of its regulon, while ERF_C_5 (*Solyc02g077370.1*) increased the expression of genes enriched in meiotic cell cycle processes, Figure 4b.

### 2.6. The Most Relevant Specific MTRs for Severe PSTVd Variant Are Coupled in a Negative Transcriptional Reprogramming

In order to identify the MTRs that are unique for the PSTVd-S23 strain and that could be correlating with the symptoms displayed by this variant, we performed an MRA and selected those that were not present for the other conditions.

Our results show that 42 TFs are acting as MTRs specifically for the severe condition, Table S1. Among those, ~30% (12 out of 42) of the unique MTRs are TFs belonging to the families of interest. They included, for instance, five members of the ERF-family ERF-1a (*Solyc05g051200.1*): RAP2-12 (*Solyc12g049560.1*), PTI6 (*Solyc06g082590.1*), TSRF1 (*Solyc09g089930.1*), and ERF_A_2 (*Solyc03g093610.1*), five bHLH-MTRs: SlbHLH011 (*Solyc01g111130.2*), bHLH130-like (*Solyc12g100140.1*), SlbHLH022 (*Solyc03g097820.1*), GBOF-1 (*Solyc06g072520.1*), and bHLH92 isoform (*Solyc09g083360.2*), as well as two MYB-type TFs: MYB1R1 (*Solyc04g005100.2*) and MYB16 (*Solyc02g088190.2*). The other remaining 70% of MTRs included diverse TFs members of multiple families including ZF-HD TFs, BZIP-TFs, and NAC-TF, among others, Table S2. We identified those that are listed in the top ten according to their regulons gene expression normalized enrichment scores (NES), which is a primary statistical value for examining gene set enrichment results. Then, we determined the MTRs predicted functional relevance.

Interestingly, these TFs are mainly acting by repressing the expression of their regulons, Figure 4. For instance, two of them were *Solyc10g077110.1* (NES = −2.9729, *p*-value = 0.003), a tf3a transcription factor TFIIIA that plays a critical role in regulating the transcription of the 5S ribosomal RNA genes by RNA polymerase III, and BZIP23, a BZIP-family *Solyc01g111580.2* (NES = −2.9667, *p*-value = 0.003) that participates in the regulation of the KEGG:00660 C5-Branched dibasic acid metabolism pathway. This particular TFIIIA has been reported to be downregulated in PSTVd-mild and S23 variants. Based on its in vitro binding capacity in *Arabidopsis thaliana* experimental tests, it is hypothesized that it could be acting as a bridge between the viroid template and the DNA polymerase II in the viroid-derived RNA replication [39]. PSTVd replicates in the host cell nuclei by an asymmetric rolling-circle mechanism, where the DNA-dependent RNA polymerase forms a complex with the TFIIIA to adopt the viroid genome as a template [40].

Notably, the presence of the bZIP family stands out at the top of the list. Those members are *Solyc01g111580.2* (NES = −2.9667, *p*-value = 0.003) which participates in the KEGG:00660 C5-Branched dibasic acid metabolism pathway, VIP1 (*Solyc06g060490.2*) (NES = 2.8289, *p*-value = 0.004) which induces chromatin assembly, DNA conformation change, nucleosome organization, and protein-DNA complex assembly, and *Solyc10g08g062960.2* (NES = −2.5241, *p*-value = 0.01) which represses cellular aldehyde metabolic process, response to desiccation, and the glyoxylate metabolic process.

**Figure 5:**
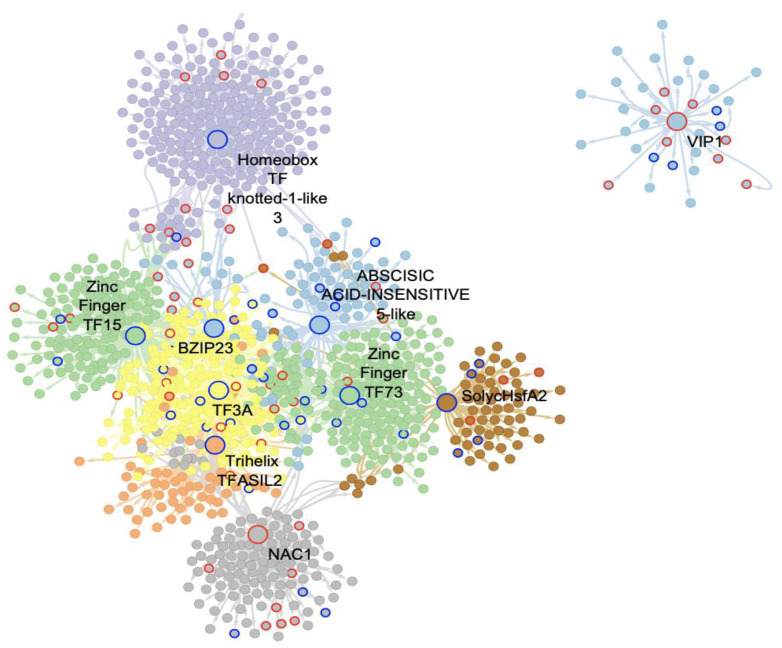
Interactome of the top unique MTRs in the severe comparison. Each node represents a gene, and the edges between nodes represent interactions between genes (regulation). Each MTR is represented by a large node, and the surrounding medium-sized nodes of the same color represent genes under its regulation (regulon). Each color represents a family: blue belongs to the bZIP family, while those green belong to the ZF-HD family. Genes in different colors are unique members of other families. The colored perimeters in blue indicate downregulation, while red depicts upregulation.

Moreover, SolycHsfA2 (*Solyc08g062960.2*), a heat shock MTR (NES = −2.5408, *p*-value = 0.01) that downregulates kinase inhibitor activity and tRNA threonyl-carbamoyl adenosine modification in the EKC/KEOPS complex, was identified. Additionally, Homeobox TF knotted-1-like 3 (*Solyc08g041820.2*), a Homeobox-MTR (NES = −2.4990, *p*-value = 0.001) downregulating the sequence-specific mRNA binding and ribose-5-phosphate isomerase activity molecular functions, was identified. Meanwhile, NAC1 (*Solyc04g009440.2*) a NAC-MTR (NES = 2.5223, *p*-value = 0.001) showed induced regulon expression, Figure 5.

### 2.7. A bHLH MTR Has a Potential Specific Role as a Microprotein (miP) in the Post-Translational Regulation of Hormone Signaling Events during Severe Infection

**Figure 6:**
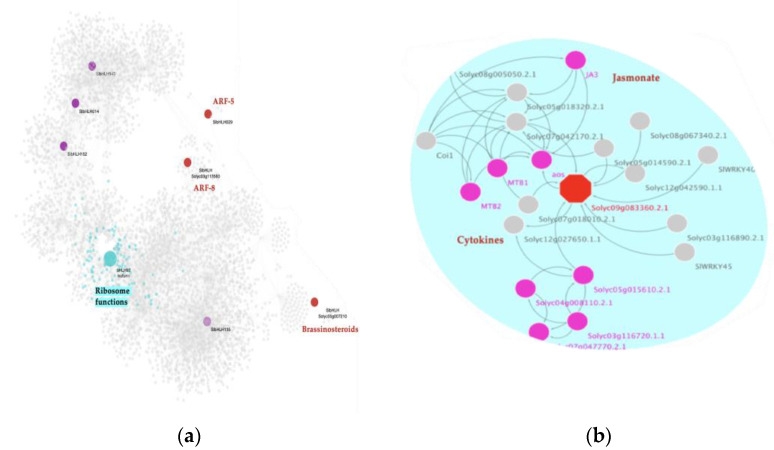
Microproteins predictive role in PSTVd severe infection: (**a**) Subnetwork depicting the presence of eight bHLH-TFs predicted as potential microproteins. Nodes in red represent those that are related to hormone-events at a protein-protein interaction level. Node in cyan represents the bHLH-TF encoding the bHLH92 isoform identified as an MTR and its corresponding regulon (small nodes in the same color). Functional enrichment of its regulon is highlighted in cyan. Nodes in purple represent the other predictive bHLH-miPs on the network; (**b**) Protein-protein interaction network of the bHLH92 isoform (*Solyc09g083360.2.1*). The hexagon node represents this microprotein, while the fuchsia nodes indicate the connected interactors related to jasmonate signaling (MYC2/JA3 bHLH transcription factor), while the others are linked to cytokines signaling events.

Using the miPfinder tool (https://github.com/DaStraub/miPFinder, accessed on 15 June 2021) we obtained a list of 39 bHLH-TFs in the tomato genome as potential miPs. Out of the 39 predicted bHLH-miPs, the following eight were found in the GRN: SlbHLH143 (*Solyc07g064040.2*), SlbHLH14 (*Solyc02g079970.2*), SlbHLH152 (*Solyc10g006510.2*), SlbHLH29 (*Solyc04g006990.2*), *Solyc05g007210.2*, *Solyc03g113560.2*, SlbHLH135 (*Solyc06g050840.2*), and the bHLH92 isoform (*Solyc09g083360.2*). When analyzing their functional role at a protein-protein interaction level, we determined that four of these biomolecules have been previously associated with diverse plant hormones, such as ARFs (*Solyc04g006990.2*, *Solyc03g113560.2*), and BRs (*Solyc05g007210.2*), as well as JA and cytokinins (CY) (bHLH92 isoform/*Solyc09g083360.2*), Figure 6a.

To gain deep insight into how these four hormones pathway-related miPs interact in each condition, we performed a mutual information algorithm analysis (26). These results suggest that bHLH miPs interactions in tomato are distinctive among the three experimental conditions, Figure S2. Moreover, this analysis shows that these four biomolecules are regulators of the PSTVd-severe condition.

Strikingly, our MRA reveals that one of those miPs, bhlh92 isoform (*Solyc09g083360.2*) (NES = 2.2725, *p*-value = 0.02), is classified as a specific MTR for the S23 infected condition. At a genetic level, this bHLH-TF is an MTR that is positively regulating downstream 122 genes involved in ribonucleoprotein complex biogenesis and translation, among other ribosome-related functions, Figure 6a. At the post-translational level, protein-protein interaction network analysis reveals that bHLH92 isoform is a 233 aa protein with a predicted function, including regulating the AOS gene, which encodes allene oxide sythase. AOS is a CYP74A subfamily member cytochrome P450 involved in the biosynthesis of jasmonic acid from lipoxygenase-derived hydroperoxides of free fatty acids. In a second interaction, it is linked to MYC2, also known as JA3 protein encoded by a BHLH TF, which we found as an MTR for the mild condition, Figure 6b. MYC2/JA3 is a transcriptional activator that binds to the G-box motif (5′-AACGTG-3′) found in the promoter of the JA-induced gene LAPA1. It acts as a negative regulator of blue light-mediated photomorphogenesis and positively regulates root growth. This gene promotes growth in response to the phytohormones ABA and JA, and binds to the G-box motif (5′-CACGTG-3′) of the RBCS-3A gene promoter. MYC2/JA3TF acts downstream of the JA receptor to orchestrate JA-mediated activation of plant responses. It has been related to positive regulation of both wound-responsive and pathogen-responsive genes through MYC2 type genes.

In summary, the specificity of the presence of these biomolecules in the mutual interaction performed using the mild and severe infected samples highlights that the differential expression and interaction of those miPs is very significant and could be relevant to pathogenesis.

### 2.8. bHLH, MYB, and ERF TF-Families Are MTRs at a Tissue-Specific Level

In order to determine the tissue-specific regulation of the MTRs identified in the global integrated analysis, we separately analyzed co-expression networks from leaf and root tissue samples. When performing the deconvolution of the network built with the integrated data (the global analysis), 69,022 interactions were obtained. While using only the expression data in the leaf transcriptomic dataset (specific tissue), a network of 29,316 interactions was obtained.

Notably, according to the global integrated analysis, in the leaf transcriptomics dataset, we identified a similar behavior of gene transcriptional regulation compared to the global regulatory network. We found that 15 out of the 21 MTRs identified in the C. vs. S23 comparison of the global analysis are conserved in genetic regulation in leaf tissue. Moreover, MYB (11) and bHLH TFs (7) are the most representative families in the C. vs. S23 comparison in those specific samples. Interestingly, in this tissue-specific analysis the top MTR is a member of the ERF family. The pathogenesis-related gene transcriptional activator, named PTI6 (*Solyc06g082590.1*) has a regulon that is enriched in the KEGG:00073-Cutin, suberine, and wax biosynthesis synaptonemal complex organization.

**Figure 7:**
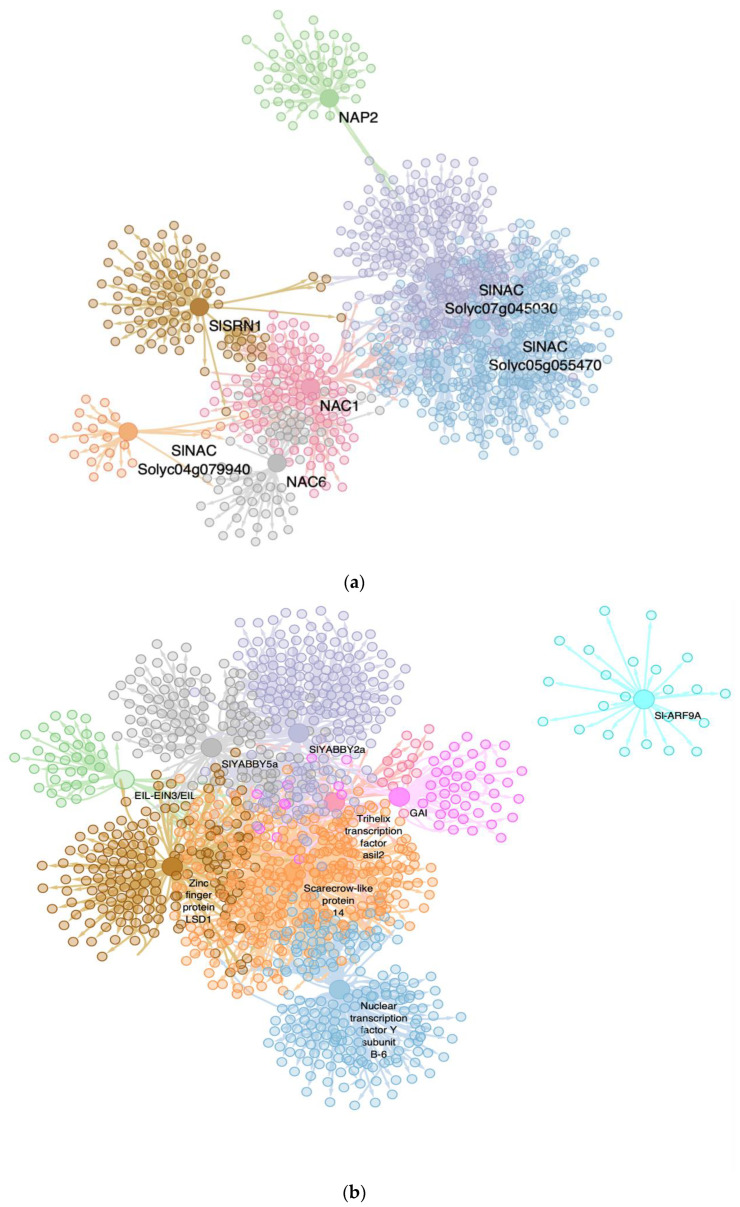
Interactomes of the C. vs. S23 conditions in leaf tissue-specific samples. Each node represents a gene, and the edges between nodes represent regulatory interactions between genes. The big, highlighted nodes with a specific color represent MTRs, while nodes of the same color surrounding these represent target genes regulated by them. (**a**): NAC family MTRs in tissue-specific (leaf) GRN: SlSRN1 (brown), NAP2 (green), SlNAC *Solyc07g045030* (purple), SlNAC *Solyc05g055470* (blue), NAC1 (pink), NAC6 (gray), SlNAC *Solyc04g079940* (orange). (**b**): Top 10 MTRs in tissue-specific (leaf) GRN: Nuclear Transcription Factor Y subunit-6 (blue), Scarecrow-like protein 14 (orange), Zinc finger protein LSD1 (brown), EIL-EIN3/EIL (green), SIYABBY5a (gray), SIYABBY2a (purple) Trihelix TF asil2 (cherry red), GAI (pink), SI-ARF9A (cyan).

As this comparison increased our insight into tissue-specific gene regulation, we noticed that all the MTRs identified for the comparison between the PSTVd variants are also significant for the healthy S23 comparison. This highlights the gene expression differences between the leaf cells infected within the two different PSTVd strains, and shows their pathogenesis mechanisms differ substantially, with the less severe strains showing similarity to the uninfected control. The top MTRs positions change according to the comparison, which in turn represents which regulons are most differentially expressed between the strains. For instance, the NAC TF family is highly relevant in the regulation by the severe variant in leaf tissue (7 members as MTRs), Figure 7a.

The TCP TF family is represented in the first two places at the top of the MTRs list for the C. vs. S23 in our global analysis. However, in specific leaf tissues, they drop to positions 24 and 28, which indicates their loss of relevance in tissue-specific regulation. Additionally, other TF families which act as MTRs appeared for the severe condition in the leaf-specific tissue analysis. These are YABBY TFs, that are related to the regulation of the initial steps of embryonic shoot apical meristem (SAM) development, and in the abaxial cell fate determination during embryogenesis and organogenesis, respectively (*Solyc07g008180.2*, *Solyc06g073920.2*) [41,42], GRAS (*Solyc11g011260.1*, *Solyc05g053090.1*) transcriptional regulators that act as repressors of the gibberellin (GA) signaling pathway. They probably act by participating in large multiprotein complexes that repress transcription of GA-inducible genes: EIL (*Solyc01g009170.2*) which binds a primary ethylene response element present in the ETHYLENE-RESPONSE-FACTOR1 promoter which then activates the transcription of this gene [43], LSD, a positive regulator of reactive oxygen-induced cell death (*Solyc08g077060.2*), NF-YB, a Histone-fold superfamily member (*Solyc06g069310.2*), Trihelix (*Solyc01g096470.2*), and ARF, an auxin-activated signaling pathway (*Solyc08g082630.2*), Figure 7b.

In contrast, when we performed the same analysis using the root transcriptomic data, no MTR was identified. This could be due to the more obvious differential gene expression among samples in the leaf microarray than in the roots transcriptomic data, where sample expression remains highly similar, Figure S3. In this sense, when discarding the root samples, the number of MTRs for all comparisons is larger in leaf-specific tissue than in the global analysis. In the C. vs. S23 123/59 MTRs (tissue-specific/global, respectively), C. vs. M 25/7, and M. vs. S23 61/21 (threshold *p* = 0.05). Overall, our results from tissue-specific networks analysis revealed conservation of the pivotal role of the bHLH, MYB, and ERF families at a tissue-specific level in tomato in response to PSTVd infection.

## 3. Discussion

Viroid pathogenesis relies upon highly complex molecular and biological processes orchestrated by both the host and the viroid genomes. This interplay of events results in asymptomatic, mild, or severe symptoms. Taking advantage of the recent time-course transcriptome analysis of the PSTVd-tomato interaction, we investigated the transcriptional regulation of genes coordinated by MTRs. It is well known that transcriptional reprogramming is governed by MTRs. In this context, it is crucial to delineate the pivotal role played by those regulators in coordinating specific regulatory networks. Moreover, the role of MTRs in the viroid pathogenesis mechanism is still unknown.

Using an *omics* approach that integrates PSTVd-infected tomato transcriptomic datasets, interactomes, and network analysis, we found the most important TFs regulating gene expression of the given phenotype of a plant-pathogen caused disease. By comparing the healthy condition (C.) with the abnormal phenotypes (S23 and M.) we determined which regulators were most likely responsible for controlling the physiological changes underlying viroid infection. Identifying the target genes of the inferred MTRs helps us to functionally characterize them for a better comprehension of how these gene expression changes are related to the different representative symptoms of the weak and severe infection phenotypes. Furthermore, comparing two different symptom phenotypes can provide us with an insight into the differences between the two phenotypes and the differential mechanisms underlying the pathogenesis caused by the two viroid strains belonging to the same pathogen species, in this case, PSTVd.

**Figure 8:**
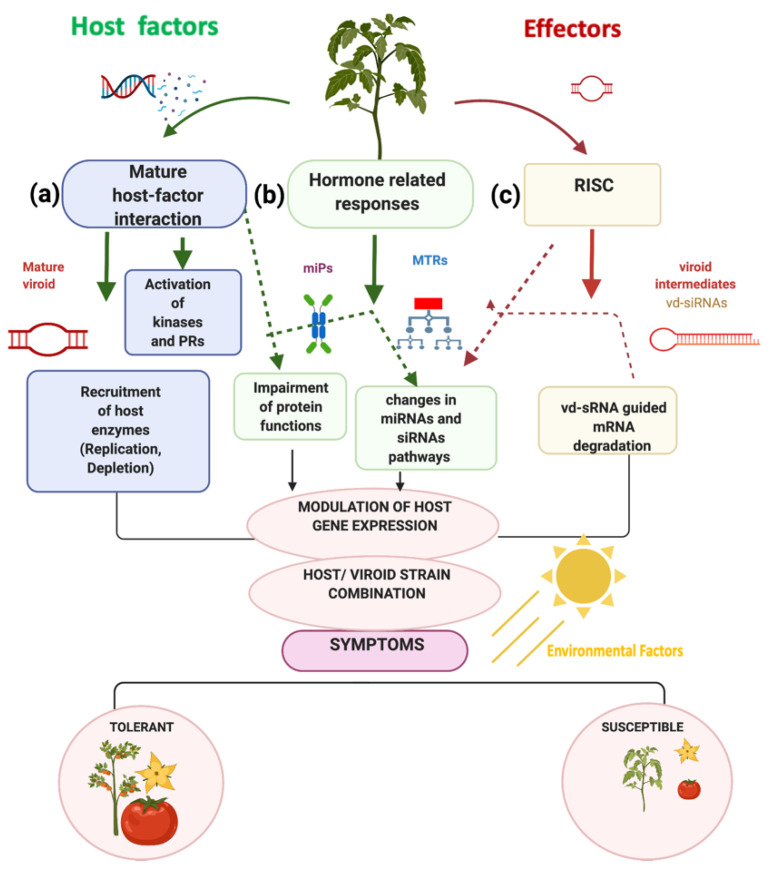
Proposed mechanism of viroid-host interplay. Symptom development is an outcome of the complex plant-pathogen interaction as a result of alterations to cell processes related to viroid RNA-mediated genetic regulation. Host factors: (**a**) description of the putative interplay of mature viroid and host factors, in which the activation of signaling and plant defense response events via kinases and pathogen related proteins (PRs) takes place, and viroid replication requires the recruitment of host enzymes; (**b**) plant hormone-related responses trigger impairment of protein functions and changes in miRNAs and siRNAs pathways; (**c**) role of viroid-derived interfering small RNAs (vd-siRNAs) that guide mRNA degradation. Dotted arrows indicate the putative links among the different RNA-mediated regulation processes. Illustration created with BioRender.com.

**Figure 9:**
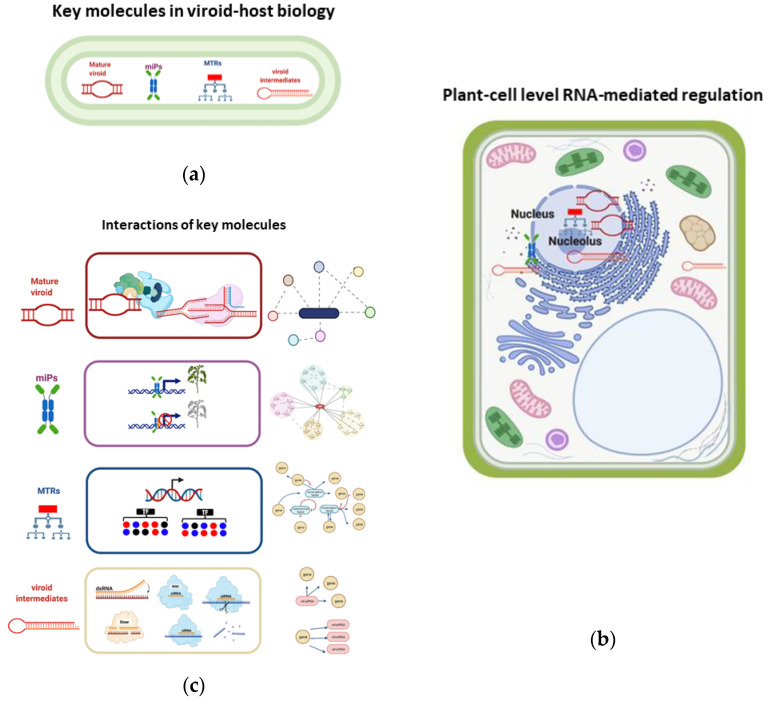
Network approaches to study viroid-host interactions. (**a**) Key molecules in viroid-host biology: mature viroid, microproteins, master transcriptional regulators, and viroid intermediates representation; (**b**) presence of mature viroid, miPs, MTRs, and vd-siRNAs as key biomolecules in plant cells and the location where they may be found; (**c**) potential network approaches to study interactomes of each type of molecule: mature viroid and its protein interactor, microProteins and their role in negative transcriptional regulation by protein-protein interactions in heterodimeric protein complexes, MTRs and their regulons in gene regulatory networks, and viroid intermediates and their mRNA targets. These strategies make it possible to have an insight into the complete mechanism of symptoms development in viroid hosts at different molecular levels. Illustration created with BioRender.com.

In a global holistic vision, the mechanism of viroid pathogenesis is highly complex, it involves the concerted interplay among host factors, as an outcome of a mature viroid/host factor interaction, activating protein kinases, and pathogenesis response proteins (PRs), as well as the recruitment of enzymes to accomplish their replication, and movement. Another mechanism relies on the activation of hormone-related responses, where microproteins and MTRs may be key molecules in the impairment of protein functions, changes in miRNAs, and siRNAs pathways, Figure 8a. Additionally, viroid intermediates from replication can be loaded into the RNA induced complex (RISC) to generate vd-siRNAs that are capable of guiding mRNA degradation, Figure 8b. Overall, those events are coupled together to modulate the host gene expression and are dependent upon the host/viroid strain combination, as well as on the participation of environmental factors. As an outcome of those highly specific and regulated events, a tolerant or a susceptible phenotype will be displayed in infected plants, Figure 8. At the plant cell level, the key molecules Figure 9a, that regulate genetic, epigenetic, and post-translational events in the nucleus, cytoplasm, and ribosomes, Figure 9b. For instance, the mature viroid interacts with RNA polymerase II and other proteins for its replication, microproteins are forming multi protein complexes to negatively regulate transcription, MTRs positively or negatively regulate downstream genes, and vd-siRNAs are pathogenic effectors that act guiding the posttranslational degradation of mRNA. All these interactions can be studied using network approaches as described in Figure 9c.

In this study, we implemented a computational strategy to identify and classify the MTRs, the functional role of their regulons, as well as potential functional microproteins under the described experimental conditions. An example of how this computational strategy differentiates the viroid strains is by identifying those key molecular responses that are conserved when comparing the PSTVd-S23 strain infected samples vs. the mock-inoculated control plants, as well as the comparison of the M. strain infected vs. the S23 infected plants. For instance, it is clearly apparent in the MYB-TFs family, in which e *kua1 isoform x1* (*Solyc08g078340.2*) MTR is common for these two comparisons. This suggests that the differential expression caused by the severe strain is very strong, and that even with an infection of a mild strain of the same viroid species, a significant difference is still observable. Moreover, this conservation of gene transcriptional regulation can be seen in the members of the ERF-TFs family. In this family, two conserved members act as MTRs when comparing C. vs. S23 and M. vs. S23 AP2/EREBP TF1 (*Solyc02g093130.1*), and ERF_C_5 (*Solyc02g077370.1*). Additionally, this result indicates that their differential expression is still appreciable when comparing samples of both the mild and severe PSTVd variants. It is also evident from the data analysis that there are five regulators with no differential expression (absent) when comparing the M. vs. S23 conditions: ERF-1a (*Solyc05g051200.1*), RAP2-12 (*Solyc12g049560.1*), PTI6 (*Solyc06g082590.1*), TSRF1 (*Solyc09g089930.1*), and ERF_A_2 (*Solyc03g093610.1*). There is also one conserved MYB-MTR that can be differentiated between the PSTVd variants. These results are in accordance with the previous PSTVd root and leaf tissue-specific transcriptomic analysis, where a similar behavior of MYB-TFs regulating gene expression as a host responsive mechanism under infection by the two variants was previously reported. These results demonstrate the potential of the approach we developed for this plant overall integrated study in which additionally we identified the potential downstream target genes of each of the MTRs.

We found that major TF families with differential expression in the S23 and M. infection are bHLH- and ERF-types which play important roles in plant defense [7,8]. In root tissue, 50% of the most strongly upregulated genes are shared in infections from both PSTVd-strains. Among the shared genes encoding the bHLH-family are TFs, which are transcriptional co-repressors of genes encoding glyoxylate reductase, NAD kinase, calcium-transporting ATPase, and chlorophyll a-b binding proteins, among other functions. Additionally, in our analysis, we included the ERF family for its abundance in plants expressing severe symptoms. Moreover, changes in the expression of TFs belonging to this family were formerly reported for viroid infection [3,7,8]. Members of this TF family are crucial in the regulation of plant development and growth, fruit ripening, defense response, and metabolism. ERFs are modulators of hormone-related processes that involve ethylene, gibberellins, cytokinins, AUX, and ABA [28,29,40,43,44,45].

We also identified those MTRs which are shared or unique for each infection condition. An example of this is observed for the MYB-TFs family group in which our analysis pointed to a member classified as MTR for the C. vs. S23 comparison, (*Solyc08g078340.2*), as well as for the M. vs. S23. Thus, this indicates that there is a high differential expression level of this MTR in severe strain infection as a differentiator between strains, since this behavior was not observed in the moderate strain. Notably, one MYB-TF was identified as a unique MTR when comparing M. vs. S23, PHR1-LIKE 1 (*Solyc05g055940.2*), and this TF may function as a differential regulator of symptom development induced by the two strains.

A totally opposite case occurs in the regulation of tomato gene expression by the bHLH TF family during viroid infection. In this TF family, MTRs identified in the two strain comparisons are all different; none of them are shared for both strains. This could suggest that the two PSTVd variants undergo transcriptional reprogramming via the same bHLH-TFs as MTRs to establish the mechanism of pathogenesis, such as SlbHLH011 (*Solyc01g111130.2*), bHLH130-like (*Solyc12g100140.1*), SlbHLH022 (*Solyc03g097820.1*), GBOF-1 (*Solyc06g072520.1*), and bHLH92 isoform (*Solyc09g083360.2*). In accordance with recent studies that identified transcription factors differentially expressed for mild and severe infection in two pepper cultivars, our results show that MYB-TFs, Homeodomain, WRKY, and heat-shock-related are specific for the severe condition [7].

Since it is impossible to describe an exhaustive list of all the TFs and their potential roles in one communication, we have performed an independent study that adopted the co-expression modularity method [45]. The objective is to characterize co-expression modules of the major TF families, including members of the bHLH, that display indispensable roles in the plant immune response. For instance, the MYC2-TF is likely to be a hub gene for the development of symptoms (24 dpi) displayed, particularly by the PSTVd-mild (M) strain [45].

In accordance with other studies that described the relevance of pathways involved in carbohydrate metabolism, we found a high enrichment of the regulons of bHLH, ERF, and unique MTRs that are predicted to participate in negative transcriptional reprogramming for the severe strain infection.

A recent study of gene expression in CBCV infected commercial hop highlighted the involvement of genes associated with the brassinosteroid pathway, as evidenced by the PPI of hub genes, such as BIM1 and BIM2, with the hop bHLH-TFs [46,47,48]. In our study, we approach the potential functional role of members of this family in the tomato genome as miPs candidates. We identified four highly relevant bHLH-TFs that are interacting to regulate the PSTVd-severe condition. To the best of our knowledge, this represents the first approach to study the role of miPs in viroid infection.

Our herein described computational strategy could be translated to study other host-pathogens interactions. However, we must consider that each viroid/host combination could lead to specific transcriptional reprogramming mechanisms, Figure 8. Regarding miPs we cannot rule out the conservancy as an interologs for other severe pospiviroid strains.

Additionally, it is noteworthy that other TFs beyond those identified in our study could be relevant and must be studied in more depth. Recently we have observed that members of a novel and poorly described TF family (PLATZ-TFs) are potential candidates as targets of regulation by multiple tomato planta macho viroid (TPMVd)-siRNAs [49]. Holistic approaches focusing on complex interactions within biological systems are now being employed to study viroid-host interactions at different regulatory levels, including epigenetic, genomic, and small RNA interference [50,51,52]. Further studies that perform *multi-omics* analysis at the level of the genome host (key molecules) and pathogen interactors (effectors, such as vd-siRNAs) will help delineate future strategies related to the use of these biomolecules. These results could serve as a basis for further analysis in the journey of refined understanding driving the development of improved management for these agronomically important diseases.

## 4. Materials and Methods

### 4.1. Designed Pipeline for the Omics Approach

For this study, we implemented a method that integrates transcriptomic data for co-expression and regulatory network analysis to give deep insight into the molecular mechanisms underlying the *Solanum lycopersicum* response to PSTVd infection, Figure 1. Our designed codes are available at https://github.com/MarcoJL9/Network-analysis-of-root-and-leaf-transcriptome-integration. First, we performed a transcriptomic integrative data analysis, followed by normalization (by rma) and data processing to generate a unique expression matrix (Steps a–b). We then used the PlantTFDB (http://planttfdb.gao-lab.org/, accessed on 25 April 2021) to obtain TF lists [20]. To perform the interactomics analysis, we used those TF-lists, and the unique expression matrix as inputs. We carried out network deconvolution analysis to have GRN as outputs using the corto algorithm (Steps c–e) [36]. Subsequently, functional enrichment analysis of the regulons under study was performed (Steps f–g). Finally, the miPs assignment and the PPI analyses were carried out (Step h).

### 4.2. Microarray Data Integration and TF Datasets for the GRN

We obtained two datasets describing transcriptome-wide effects of PSTVd tomato infection. Microarray expression datasets of root (GSE111736) and leaf (GSE106912) tomato transcriptome in mild and severe PSTVd infection were obtained from the NCBI GEO database (https://www.ncbi.nlm.nih.gov/gds, accessed on 7 May 2021). Those studies consisted of experiments employing a time-course in three stages using Control (C.), PSTVd-mild (M.), and PSTVd-severe (S23) samples. The time course is as follows: early symptoms (17 dpi) samples, complete symptoms (24 dpi), and recovery (49 dpi). In total, 26 root and 27 leaf samples were separately analyzed [7,8]. In our study, data integration of both transcriptome datasets was performed to obtain a unique expression matrix. Data integration consisted in merging datasets in order to have only the variant infection treatments of both tissues, [53,54,55]. A mean value was calculated for each repeated gene on the dataset, then the matrix was normalized using the MRA function of the affy package [50]. Our integrated expression matrix is composed of a total of 53 comparable samples in three different conditions: C:18 samples 9 from each tissue; PSTVd-M: 18 samples, 9 from each tissue; and PSTVd-S23: 17 samples 9 and 8 from each tissue.

### 4.3. Identification of Biological Communities in the Gene Regulatory Network

To approach the study of the biological significance of the genes in the regulatory networks, they were grouped according to their role in biological communities. This analysis was estimated using the Louvain method. The Louvain method is a greedy optimization method that optimizes the modularity of a partition of the network [37]. These metrics were implemented using the NetworkX package in Python scripts [56]. Then, functional enrichment analyses were assessed for each estimated community using the over-representation analysis of the functional assignment terms, including GO: terms and KEGG pathways. The estimated communities that do not have functional relevance were discarded. The g: Profiler R package was employed for data analysis [57].

### 4.4. Identification of bHLH-miPs and Their Functional Assignment

For the miPs classification, we employed the miPFinder program (https://github.com/DaStraub/miPFinder, accessed on 5 May 2021) using the bHLH TF list from the PlantTFDB to determine which members are potential candidates for encoding functional microproteins. Then, protein-protein interaction analyses were performed on each estimated bHLH-miP using the String database (https://string-db.org/, accessed on 16 June 2021). The miPs that do not have functional relevance linked to hormone pathways were discarded. For each miP-ppi network visualization, the Cytoscape environment was used [58].

### 4.5. Deconvolution of the Gene Regulatory Network

For the inference of the coexpression networks the corto algorithm with default parameters, freely available on the CRAN repository of R packages, was used. The corto package is a co-expression-based tool that infers GRNs using a TFs list with their targets, and an expression matrix data set [36]. In brief, corto uses a combination of Spearman correlation and Data Processing Inequality (DPI), adding bootstrapping to evaluate the significant edges, removing indirect interactions. As an input, we used the integrated expression matrix and the TF list described in the Section 4.2. For this analysis, a *p*-value = 1 × 10^−8^ was used as a cut-off, and 100 bootstraps were carried out. Subsequently, a single network for the integrated expression matrix was obtained, generating, as a result, an output file in which an inferred enriched GRN is contained. corto

### 4.6. Identification of the Master Transcriptional Regulators (MTRs) and Their Functional Relevance in the Three Experimental Conditions

Master regulator analyses were performed by comparing infected and mock samples with the corto algorithm, using default parameters and the coexpression network derived from the integrated expression dataset [36]. Using this R package, we inferred the MTRs in the transition between two given conditions. The MRA function implicit in the package calculates the enrichment of each TF-centered network in a user-selected signature (a list specifying which samples correspond to each condition), provided as two gene expression matrices (e.g., diseased tissue vs. control). For this study, we performed an MRA for each comparison among conditions; C vs. S23.; C. vs. M; M vs. S23. In order to determine the master transcriptional regulators, we compared those three different conditions using a *p*-value of 0.05 as a cut-off. As an output, we obtain a list of master regulators ordered by their Normalized Enrichment Score (NES), which is a measurement of how different the expression of a given regulon is when comparing two conditions. A combined value for the coexpression network is generated by weighting every gene likelihood in the network using the corto algorithm, providing a final NES, which is positive if the regulon is upregulated by the infection, and negative if it is downregulated. Finally, functional enrichment analyses were assessed to each regulon estimated using the over-representation analysis of the functional assignment terms, including GO: terms and the KEGG pathways. The g: Profiler R package was employed for data analysis [57]. The Cytoscape environment was used for each subnetwork visualization [58].

## 5. Conclusions

Gene regulatory networks of mild and S23-severe PSTVd variants from transcriptomics analysis revealed that specific bHLH, MYB, and ERF MTRs are regulating genes among experimental treatments. By performing network analysis and functional enrichment we determined those MTRs and their targets that participate in molecular mechanisms underlying distinct and shared biological processes. Notably, the identification of bHLH TFs in the networks, and the elucidation of the important function they play among treatments, revealed them as potential miPs related to ARFs, BR, and JA hormones involved in post-translational regulation. Moreover, the important role of one of these molecules, bhlh92 isoform (*Solyc09g083360.2*), is highlighted as MTR for the severe PSTVd strain. Our study represents a pioneering attempt to identify and classify PSTVd-responsive tomato MTRs and provides valuable information about their involvement in the development of viroid pathogenesis in this host. Altogether, our results lay a foundation for further research on the PSTVd and host genome interaction, providing evidence for the identification of potential key genes that influence symptoms during development in tomato plants.

## Data Availability

Datasets: Microarray from roots samples: https://www.ncbi.nlm.nih.gov/geo/query/acc.cgi?acc=GSE111736, accessed on 7 May 2021. Microarray from leaf samples: https://www.ncbi.nlm.nih.gov/geo/query/acc.cgi?acc=GSE106912, accessed on 7 May 2021. Integrated expression matrix from both tissues: https://github.com/MarcoJL9/Network-analysis-of-root-and-leaf-transcriptome-integration. Code: https://github.com/MarcoJL9/Network-analysis-of-root-and-leaf-transcriptome-integration.

## Supplementary Materials

The following supporting information can be downloaded at: https://www.mdpi.com/article/10.3390/ijms23115983/s1.

## Appendix A

List of the gene communities found in the bHLH, ERF, and MYB interactomes. https://docs.google.com/spreadsheets/d/1kpS7nP7MtCda9TIFZX7xboo9lDjBcPz7qABIOQHFROA/edit#gid=0.

## Appendix B

Functional enrichment analysis of the identified regulons of the MTRs.

## References

[1] Diener T.O. (1971). Potato Spindle Tuber “Virus”. Virology.

[2] Katsarou K., Adkar-Purushothama C.R., Tassios E., Samiotaki M., Andronis C., Lisón P., Nikolaou C., Perreault J.-P., Kalantidis K. (2022). Revisiting the Non-Coding Nature of Pospiviroids. Cells.

[3] Aviña-Padilla K., Rivera-Bustamante R., Kovalskaya N., Hammond R. (2018). Pospiviroid Infection of Tomato Regulates the Expression of Genes Involved in Flower and Fruit Development. Viruses.

[4] Wang Y., Shibuya M., Taneda A., Kurauchi T., Senda M., Owens R.A., Sano T. (2011). Accumulation of Potato Spindle Tuber Viroid-Specific Small RNAs Is Accompanied by Specific Changes in Gene Expression in Two Tomato Cultivars. Virology.

[5] Owens R.A., Tech K.B., Shao J.Y., Sano T., Baker C.J. (2012). Global Analysis of Tomato Gene Expression during Potato Spindle Tuber Viroid Infection Reveals a Complex Array of Changes Affecting Hormone Signaling. Mol. Plant Microbe Interact..

[6] Itaya A., Matsuda Y., Gonzales R.A., Nelson R.S., Ding B. (2002). Potato Spindle Tuber Viroid Strains of Different Pathogenicity Induces and Suppresses Expression of Common and Unique Genes in Infected Tomato. Mol. Plant Microbe Interact..

[7] Więsyk A., Iwanicka-Nowicka R., Fogtman A., Zagórski-Ostoja W., Góra-Sochacka A. (2018). Time-Course Microarray Analysis Reveals Differences between Transcriptional Changes in Tomato Leaves Triggered by Mild and Severe Variants of Potato Spindle Tuber Viroid. Viruses.

[8] Góra-Sochacka A., Więsyk A., Fogtman A., Lirski M., Zagórski-Ostoja W. (2019). Root Transcriptomic Analysis Reveals Global Changes Induced by Systemic Infection of *Solanum lycopersicum* with Mild and Severe Variants of Potato Spindle Tuber Viroid. Viruses.

[9] Hadjieva N., Apostolova E., Baev V., Yahubyan G., Gozmanova M. (2021). Transcriptome Analysis Reveals Dynamic Cultivar-Dependent Patterns of Gene Expression in Potato Spindle Tuber Viroid-Infected Pepper. Plants.

[10] Wang Y., Wu J., Qiu Y., Atta S., Zhou C., Cao M. (2019). Global Transcriptomic Analysis Reveals Insights into the Response of “Etrog” Citron (*Citrus medica* L.) to Citrus Exocortis Viroid Infection. Viruses.

[11] Tessitori M., Maria G., Capasso C., Catara G., Rizza S., De Luca V., Catara A., Capasso A., Carginale V. (2007). Differential Display Analysis of Gene Expression in Etrog Citron Leaves Infected by Citrus Viroid III. Biochim. Biophys. Acta.

[12] Herranz M.C., Niehl A., Rosales M., Fiore N., Zamorano A., Granell A., Pallas V. (2013). A Remarkable Synergistic Effect at the Transcriptomic Level in Peach Fruits Doubly Infected by Prunus Necrotic Ringspot Virus and Peach Latent Mosaic Viroid. Virol. J..

[13] Kappagantu M., Bullock J.M., Nelson M.E., Eastwell K.C. (2017). Hop Stunt Viroid: Effect on Host (*Humulus lupulus*) Transcriptome and Its Interactions with Hop Powdery Mildew (*Podospheara macularis*). Mol. Plant Microbe Interact..

[14] Xia C., Li S., Hou W., Fan Z., Xiao H., Lu M., Sano T., Zhang Z. (2017). Global Transcriptomic Changes Induced by Infection of Cucumber (*Cucumis sativus* L.) with Mild and Severe Variants of Hop Stunt Viroid. Front. Microbiol..

[15] Mishra A., Kumar A., Mishra D., Nath V., Jakše J., Kocábek T., Killi U., Morina F., Matoušek J. (2018). Genome-Wide Transcriptomic Analysis Reveals Insights into the Response to Citrus Bark Cracking Viroid (CBCVd) in Hop (*Humulus lupulus* L.). Viruses.

[16] Kimura S., Sinha N. (2008). Tomato (*Solanum lycopersicum*): A Model Fruit-Bearing Crop. CSH Protoc..

[17] Quinet M., Angosto T., Yuste-Lisbona F.J., Blanchard-Gros R., Bigot S., Martinez J.P., Lutts S. (2019). Tomato Fruit Development and Metabolism. Front. Plant Sci..

[18] Maureira F., Rajagopalan K., Stöckle C.O. (2022). Evaluating Tomato Production in Open-Field and High-Tech Greenhouse Systems. J. Clean. Prod..

[19] FAO FAOSTAT Food and Agricultural Organization Statistics. http://faostat.fao.org/site/567/DesktopDefault.aspx?PageID=567#ancor.

[20] Jin J., Tian F., Yang D.-C., Meng Y.-Q., Kong L., Luo J., Gao G. (2016). PlantTFDB 4.0: Toward a Central Hub for Transcription Factors and Regulatory Interactions in Plants. Nucleic Acids Res..

[21] Guzzi P.H., Mercatelli D., Ceraolo C., Giorgi F.M. (2020). Master Regulator Analysis of the SARS-CoV-2/Human Interactome. J. Clin. Med..

[22] Hernández-Lemus E., Tovar H. (2020). Networks of Transcription Factors. Genome Plasticity in Health and Disease.

[23] Galindo A.J. (1982). Etiology of Planta Macho, a Viroid Disease of Tomato. Phytopathology.

[24] Zhou M., Memelink J. (2016). Jasmonate-Responsive Transcription Factors Regulating Plant Secondary Metabolism. Biotechnol. Adv..

[25] Catinot J., Huang J.-B., Huang P.-Y., Tseng M.-Y., Chen Y.-L., Gu S.-Y., Lo W.-S., Wang L.-C., Chen Y.-R., Zimmerli L. (2015). Ethylene Response Factor 96 Positively RegulatesArabidopsisresistance to Necrotrophic Pathogens by Direct Binding to GCC Elements of Jasmonate—And Ethylene-Responsive Defence Genes. Plant Cell Environ..

[26] Feller A., Machemer K., Braun E.L., Grotewold E. (2011). Evolutionary and Comparative Analysis of MYB and bHLH Plant Transcription Factors. Plant J..

[27] Rehman S., Mahmood T. (2015). Functional Role of DREB and ERF Transcription Factors: Regulating Stress-Responsive Network in Plants. Acta Physiol. Plant.

[28] Gutterson N. (2004). Regulation of Disease Resistance Pathways by AP2/ERF Transcription Factors. Curr. Opin. Plant Biol..

[29] Sacharowski S.P., Gratkowska D.M., Sarnowska E.A., Kondrak P., Jancewicz I., Porri A., Bucior E., Rolicka A.T., Franzen R., Kowalczyk J. (2015). SWP73 Subunits of Arabidopsis SWI/SNF Chromatin Remodeling Complexes Play Distinct Roles in Leaf and Flower Development. Plant Cell.

[30] Yuan L. (2020). Clustered ERF Transcription Factors: Not All Created Equal. Plant Cell Physiol..

[31] Staudt A., Wenkel S. (2010). Regulation of Protein Function by “MicroProteins”. EMBO Rep..

[32] Eguen T., Straub D., Graeff M., Wenkel S. (2015). MicroProteins: Small Size—Big Impact. Trends Plant Sci..

[33] Aviña-Padilla K., Ramírez-Rafael J.A., Herrera-Oropeza G.E., Muley V.Y., Valdivia D.I., Díaz-Valenzuela E., García-García A., Varela-Echavarría A., Hernández-Rosales M. (2021). Evolutionary Perspective and Expression Analysis of Intronless Genes Highlight the Conservation of Their Regulatory Role. Front. Genet..

[34] Yang C., Huang S., Zeng Y., Liu C., Ma Q., Pruneda-Paz J., Kay S.A., Li L. (2021). Two bHLH Transcription Factors, bHLH48 and bHLH60, Associate with Phytochrome Interacting Factor 7 to Regulate Hypocotyl Elongation in Arabidopsis. Cell Rep..

[35] Ahmad Z. (2021). A Big Role for MicroProteins in Preventing Premature Floral Transition in the Shoot Meristem. Plant Physiol..

[36] Mercatelli D., Lopez-Garcia G., Giorgi F.M. (2020). Corto: A Lightweight R Package for Gene Network Inference and Master Regulator Analysis. Bioinformatics.

[37] Blondel V.D., Guillaume J.-L., Lambiotte R., Lefebvre E. (2008). Fast Unfolding of Communities in Large Networks. J. Stat. Mech..

[38] Wang Y., van der Hoeven R., Nielsen R., Mueller L., Tanksley S. (2005). Characteristics of the tomato nuclear genome as determined by sequencing undermethylated EcoRI digested fragments. Theor. Appl. Genet..

[39] Layat E., Cotterell S., Vaillant I., Yukawa Y., Tutois S., Tourmente S. (2012). Transcript Levels, Alternative Splicing and Proteolytic Cleavage of TFIIIA Control 5S RRNA Accumulation during Arabidopsis Thaliana Development. Plant J..

[40] Dissanayaka Mudiyanselage S., Qu J., Tian N., Jiang J., Wang Y. (2018). Potato Spindle Tuber Viroid RNA-Templated Transcription: Factors and Regulation. Viruses.

[41] Liu M., Chen Y., Chen Y., Shin J.-H., Mila I., Audran C., Zouine M., Pirrello J., Bouzayen M. (2018). The Tomato Ethylene Response Factor Sl-ERF.B3 Integrates Ethylene and Auxin Signaling via Direct Regulation of Sl-Aux/IAA27. New Phytol..

[42] Srivastava R., Kumar R. (2019). The Expanding Roles of APETALA2/Ethylene Responsive Factors and Their Potential Applications in Crop Improvement. Brief. Funct. Genom..

[43] Fujimoto S.Y., Ohta M., Usui A., Shinshi H., Ohme-Takagi M. (2000). Arabidopsis Ethylene-Responsive Element Binding Factors Act as Transcriptional Activators or Repressors of GCC Box-Mediated Gene Expression. Plant Cell.

[44] Peluso J., Delidow B., Lynch J., White B. (1991). Follicle-stimulating hormone and insulin regulation of 17 beta-estradiol secretion and granulosa cell proliferation within immature rat ovaries maintained in perifusion culture. Endocrinology.

[45] Solano R., Stepanova A., Chao Q., Ecker J. (1998). Nuclear events in ethylene signaling: A transcriptional cascade mediated by ETHYLENE-INSENSITIVE3 and ETHYLENE-RESPONSE-FACTOR1. Genes Dev..

[46] Gai S., Zhang Y., Liu C., Zhang Y., Zheng G. (2013). Transcript Profiling of *Paoenia ostii* during Artificial Chilling Induced Dormancy Release Identifies Activation of GA Pathway and Carbohydrate Metabolism. PLoS ONE.

[47] Sukumari Nath V., Kumar Mishra A., Kumar A., Matoušek J., Jakše J. (2019). Revisiting the Role of Transcription Factors in Coordinating the Defense Response against Citrus Bark Cracking Viroid Infection in Commercial Hop (*Humulus lupulus* L.). Viruses.

[48] Márquez-Molins J., Villalba-Bermell P., Corell-Sierra J., Pallás V., Gómez G. (2022). Integrative Time-Scale and Multi-Omic Analysis of Host-Responses to Hop Stunt Viroid Infection. bioRxiv.

[49] Abrahamiam P. (2021). Analysis of SRNA Seq Data from TPMVd-Infected Tomato Plants.

[50] Reverter A., Chan E.K.F. (2008). Combining Partial Correlation and an Information Theory Approach to the Reversed Engineering of Gene Co-Expression Networks. Bioinformatics.

[51] Aviña-Padilla K., Zambada-Moreno O., Herrera-Oropeza G.E., Jimenez-Limas M.A., Hammond R., Hudson M., Hernández-Rosales M. (2022). Dynamic co-expression network analysis of root PSTVd-infected tomato reveals the interplay of bHLH TFs. Int. J. Mol. Sci..

[52] Cheng C.W., Beech D.J., Wheatcroft S.B. (2020). Advantages of CEMiTool for Gene Co-Expression Analysis of RNA-Seq Data. Comput. Biol. Med..

[53] Ghazalpour A., Doss S., Zhang B., Wang S., Plaisier C., Castellanos R., Brozell A., Schadt E.E., Drake T.A., Lusis A.J. (2006). Integrating genetic and network analysis to characterize genes related to mouse weight. PLoS Genet..

[54] Bonin-Andresen M., Smiljanovic B., Stuhlmüller B., Sörensen T., Grützkau A., Häupl T. (2018). Bedeutung von Big Data für die molekulare Diagnostik. Z. Rheumatol..

[55] Ovens K., Eames B.F., McQuillan I. The impact of sample size and tissue type on the reproducibility of gene co-expression networks. Proceedings of the 11th ACM International Conference on Bioinformatics, Computational Biology and Health Informatics 2020.

[56] Hagberg A., Swart P., Chult D.S. (2008). Exploring Network Structure, Dynamics, and Function Using Networkx.

[57] Kolberg L., Raudvere U., Kuzmin I., Vilo J., Peterson H. (2020). gprofiler2—An R Package for Gene List Functional Enrichment Analysis and Namespace Conversion Toolset g:Profiler. F1000Research.

[58] Shannon P. (2003). Cytoscape: A Software Environment for Integrated Models of Biomolecular Interaction Networks. Genome Res..

