# Supplementary material for: Insights into the Transcriptional Reprogramming in Tomato Response to PSTVd Variants Using Network Approaches
Source: Int J Mol Sci. 2022 May 26;23(11):5983. doi: 10.3390/ijms23115983 (PMC9181013; doi:10.3390/ijms23115983)
Supplement: Supplementary file 1 [file ijms-23-05983-s001.zip › ijms-1728572-supplementary.pdf]

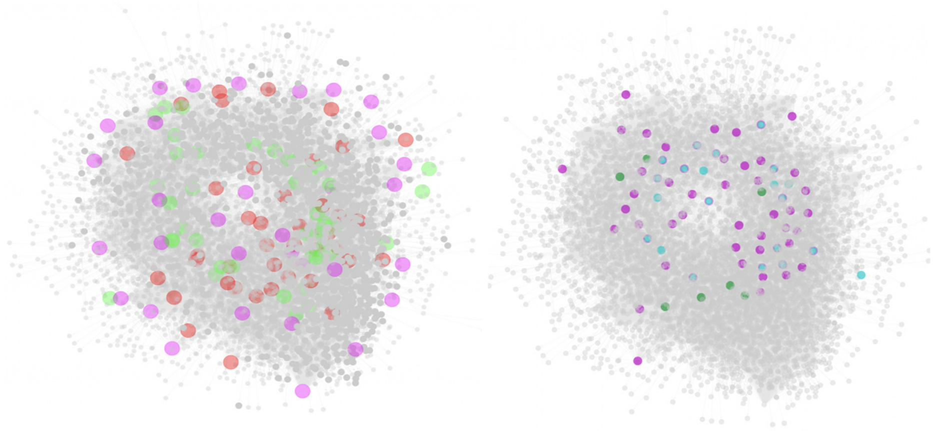

(a) **(b)**

Figure S1. Gene Regulatory Global Networks. Each node represents a gene; the edges among nodes depict the regulatory action of one node on another(s). a): GRN for the integrated data is presented on the left; the most prominent nodes highlighted in different colors indicate all the TFs of the families of interest in this study that are present in the global network; bHLH-TF (pink), MYB-TF (green), and ERF-TF (red). b): GRN for only leaf data is presented on the right; the most prominent nodes highlighted in different colors indicate all the TFs of the families of interest in this study that are present in the global network; bHLH-TF (purple), MYB-TF (green), and ERF-TF (cyan).

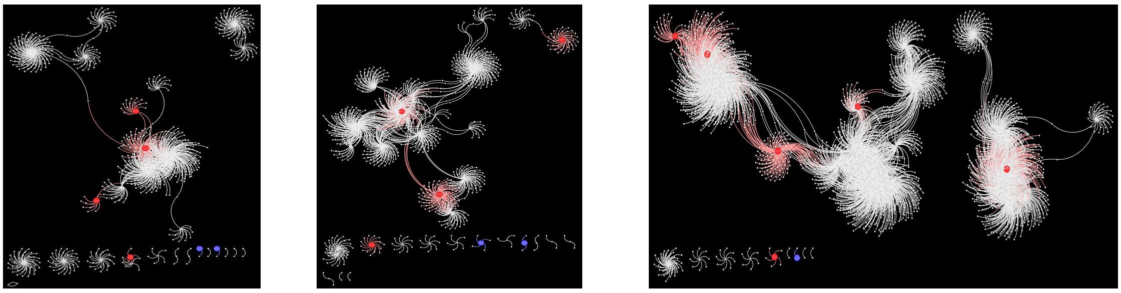

(**a**) (**b**) (**c**)

Figure S2. Mutual interaction networks. miP-molecule interactions among the different conditions from left to right: a): C. samples, b): PSTVd-Mn, and c): PSTVd-S23. Each node represents a gene, the edges between nodes depict putative interactions among them(regulation). Nodes highlighted in red color represent the hub bHLH-miPs, while the edges of the same color represent their predicted interactions with other molecules. Nodes in blue color represent bHLH-miPs that are not hub genes.

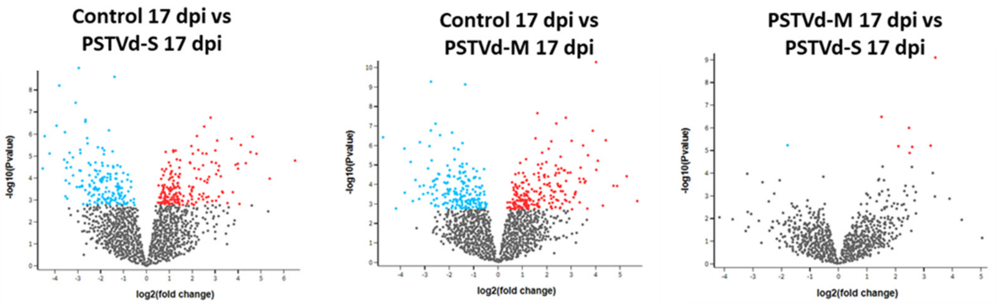

(a)

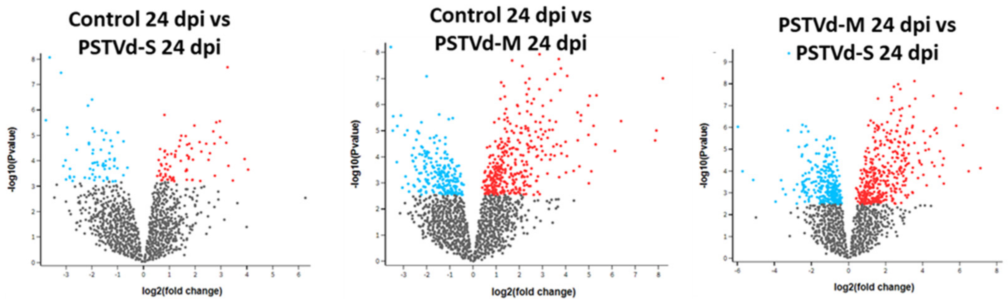

(b)

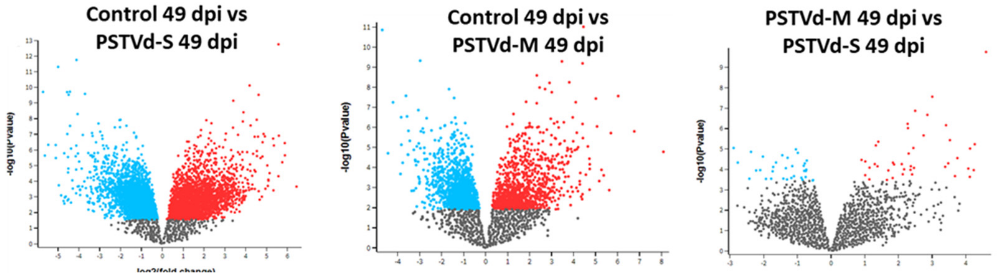

(c)

Figure S3. Comparative analysis of gene expression among treatments in tomato roots. Volcano plots depicting the differential expression among C., PSTVd-S23 and PSTVd-M strains in the three infection stages. a): early (17 dpi), b); complete symptoms (24 dpi), and c): recovery (49 dpi). Each dot represents one gene, red colored dots denote significantly differentially expressed genes of upregulated genes that achieved an adjusted p-value <0.05, while blue colored dots represent downregulated genes.

Table S1 List of all the MTRs significant for the three conditions

Gene ID	TF family	NES	P value	Contition
Solyc01g008230.2	TCP	-3.49020967	0.00048264	C vs S
Solyc06g070900.2	TCP	-3.40100679	0.00067138	C vs S
Solyc01g110490.2	C3H	-3.15758282	0.00159083	C vs S
Solyc01g087990.2	MADS-box	3.11312826	0.00185116	C vs S
Solyc05g051200.1	ERF	2.98168476	0.00286667	C vs S
Solyc10g077110.1	tf3a	-2.97292849	0.00294973	C vs S
Solyc01g111580.2	BZIP	-2.96679446	0.00300922	C vs S
Solyc02g077370.1	ERF	2.94342498	0.00324602	C vs S
Solyc05g053090.1	GRAS	2.91426618	0.00356526	C vs S

Solyc01g111130.2	bHLH	-2.87776345	0.00400505	C vs S
Solyc06g060490.2	BZIP	2.82899978	0.00466937	C vs S
Solyc11g072470.1	LOB	2.79901433	0.00512589	C vs S
Solyc02g093130.1	ERF	-2.75096964	0.00594191	C vs S
Solyc12g049560.1	ERF	-2.72200831	0.00648865	C vs S
Solyc09g009100.2	Hsf	-2.70420572	0.00684679	C vs S
Solyc04g074990.2	ZF-HD	-2.64713109	0.00811779	C vs S
Solyc06g069760.2	DOF	-2.5751669	0.01001917	C vs S
Solyc01g109880.2	BZIP	2.55792504	0.01052988	C vs S
Solyc08g062960.2	Hsf	-2.54085675	0.01105812	C vs S
Solyc12g100140.1	bHLH	-2.52716787	0.01149865	C vs S
Solyc10g081350.1	BZIP	-2.52416514	0.01159734	C vs S
Solyc04g009440.2	NAC	2.52231799	0.01165843	C vs S
Solyc02g076810.2	Trihelix	-2.50120693	0.01237708	C vs S
Solyc08g041820.2	Homeobox	-2.49909905	0.01245095	C vs S
Solyc06g082590.1	ERF	2.46587721	0.01366782	C vs S
Solyc12g008660.1	ZF-HD	-2.45771029	0.01398259	C vs S
Solyc04g005100.2	MYB	2.45739237	0.01399497	C vs S
Solyc02g088190.2	MYB	-2.45231432	0.01419406	C vs S
Solyc10g083510.1	GRF	-2.42811586	0.01517749	C vs S
Solyc08g078340.2	MYB	-2.40532948	0.01615789	C vs S
Solyc06g073730.1	EIL	2.38820768	0.01693077	C vs S
Solyc08g076930.1	bHLH	2.37784856	0.01741398	C vs S
Solyc06g008200.2	G2-like	2.36462605	0.01804828	C vs S
Solyc07g045030.2	NAC	2.33199841	0.01970078	C vs S
Solyc03g097820.1	bHLH	2.33095641	0.01975566	C vs S
Solyc06g072520.1	bHLH	2.33095641	0.01975566	C vs S
Solyc09g089930.1	ERF	2.3162817	0.02054289	C vs S
Solyc02g093050.2	WRKY	2.31030967	0.02087102	C vs S
Solyc08g077060.2	LSD	-2.30453155	0.02119282	C vs S
Solyc07g065500.1	NF-YB	-2.29297692	0.02184933	C vs S
Solyc12g056790.1	NAC	2.2770349	0.02278414	C vs S
Solyc09g083360.2	bHLH	2.2725908	0.02305085	C vs S
Solyc03g110860.2	NF-YC	-2.24330826	0.02487694	C vs S
Solyc01g014850.2	C3H	-2.20251292	0.0276291	C vs S
Solyc02g090820.2	Hsf	2.17805822	0.0294017	C vs S
Solyc01g090840.2	C2H2	2.16355787	0.0304983	C vs S
Solyc12g088390.1	C2H2	2.16355787	0.0304983	C vs S
Solyc06g072040.1	CONSTANS	-2.15698809	0.03100659	C vs S
Solyc07g020710.2	CPP	2.08785585	0.03681084	C vs S
Solyc05g009660.2	bZIP	-2.0800722	0.03751891	C vs S
Solyc01g096700.2	MYB_related	-2.0768131	0.03781881	C vs S
Solyc02g085610.2	GBF	-2.07568583	0.03792302	C vs S
Solyc11g072500.1	DOF	-2.07481109	0.03800405	C vs S
Solyc02g084230.1	BBR-BCP	-2.06519596	0.03890446	C vs S

Solyc02g080890.2	WRKY	2.06172495	0.03923393	C vs S
Solyc03g093610.1	ERF	2.02249216	0.04312553	C vs S
Solyc02g081120.2	knotted-like	-2.01895108	0.0434923	C vs S
Solyc07g062710.2	BZIP	-1.96510699	0.04940186	C vs S
Solyc10g055760.1	NAC	1.96456755	0.04946431	C vs S
Solyc07g052760.1	GeBP	-2.30254424	0.0213	C vs M
Solyc01g097330.2	bZIP	2.26359513	0.0236	C vs M
Solyc09g074640.2	ZF-HD	-2.16881365	0.0301	C vs M
Solyc06g069760.2	DOF	-2.13768943	0.0325	C vs M
Solyc09g009100.2	Hsf	-2.05470047	0.0399	C vs M
Solyc11g044560.1	BZIP	-2.02853796	0.0425	C vs M
Solyc03g098320.2	MYB	-2.00696852	0.0448	C vs M
Solyc01g008230.2	TCP	-3.32513881	0.000884	M vs S
Solyc01g109700.2	bHLH	-3.13520645	0.00172	M vs S
Solyc06g070900.2	TCP	-2.97283719	0.00295	M vs S
Solyc01g087990.2	MAD-box	2.8936067	0.00381	M vs S
Solyc11g072470.1	LOB	2.814547	0.00488	M vs S
Solyc05g053090.1	GRAS	2.80120068	0.00509	M vs S
Solyc04g005610.2	NAC	2.74520258	0.00605	M vs S
Solyc01g109880.2	BZIP	2.63818413	0.00834	M vs S
Solyc02g093130.1	ERF	-2.54113726	0.011	M vs S
Solyc02g077370.1	ERF	2.53305973	0.0113	M vs S
Solyc03g120890.2	GATA	-2.44145193	0.0146	M vs S
Solyc08g077060.2	LSD1	-2.26162577	0.0237	M vs S
Solyc07g045030.2	NAC	2.23815203	0.0252	M vs S
Solyc08g078340.2	MYB	-2.13376081	0.0329	M vs S
Solyc03g097120.2	Hsf	2.12271572	0.0338	M vs S
Solyc04g074990.2	ZF-HD	-2.11929425	0.0341	M vs S
Solyc08g076930.1	bHLH	2.04815701	0.0405	M vs S
Solyc07g065500.1	MYB	-2.03118794	0.0422	M vs S
Solyc06g069430.2	MAD-box	2.01977398	0.0434	M vs S
Solyc06g073730.1	MYB	2.01312508	0.0441	M vs S
Solyc05g055940.2	NF-YB	1.9880456	0.0468	M vs S

Table S2. Top 10 MTRs significant for Severe condition that do not belong to the interest families.

Gene ID	Family	NES	pValue
Solyc01g110490.2	ZF-HD	-3.15758282	0.00159083
Solyc10g077110.1	tf3a	-2.97292849	0.00294973
Solyc01g111580.2	BZIP	-2.96679446	0.00300922
Solyc06g060490.2	BZIP	2.82899978	0.00466937
Solyc08g062960.2	Hsf	-2.54085675	0.01105812
Solyc10g081350.1	BZIP	-2.52416514	0.01159734
Solyc04g009440.2	NAC	2.52231799	0.01165843
Solyc02g076810.2	Trihelix	-2.50120693	0.01237708

Solyc08g041820.2	Homeobox	-2.49909905	0.01245095
Solyc12g008660.1	ZF-HD	-2.45771029	0.01398259
